# Spin-labelled mechanically interlocked molecules as models for the interpretation of biradical EPR spectra†

**DOI:** 10.1039/d1sc01462e

**Published:** 2021-05-14

**Authors:** Lorenzo Gualandi, Paola Franchi, Elisabetta Mezzina, Stephen M. Goldup, Marco Lucarini

**Affiliations:** a Department of Chemistry “Giacomo Ciamician”, University of Bologna Via San Giacomo 11 Bologna Italy marco.lucarini@unibo.it; b Department of Chemistry, University of Southampton University Road, Highfield Southampton UK s.goldup@soton.ac.uk

## Abstract

Biradical spin probes can provide detailed information about the distances between molecules/regions of molecules because the through-space coupling of radical centres, characterised by *J*, is strongly distance dependent. However, if the system can adopt multiple configurations, as is common in supramolecular complexes, the shape of the EPR spectrum is influenced not only by *J* but also the rate of exchange between different states. In practice, it is often hard to separate these variables and as a result, the effect of the latter is sometimes overlooked. To demonstrate this challenge unequivocally we synthesised rotaxane biradicals containing nitronyl nitroxide units at the termini of their axles. The rotaxanes exchange between the available biradical conformations more slowly than the corresponding non-interlocked axles but, despite this, in some cases, the EPR spectra of the axle and rotaxane remain remarkably similar. Detailed analysis allowed us to demonstrate that the similar EPR spectral shapes result from different combinations of *J* and rates of conformational interconversion, a phenomenon suggested theoretically more than 50 years ago. This work reinforces the idea that thorough analysis must be performed when interpreting the spectra of biradicals employed as spin probes in solution.

## Abstract 2

Using a rotaxane biradical, we unambiguously demonstrate the need consider both *J* and rate of conformational interconversion carefully when interpreting the spectra of such systems when they are employed as spin probes in solution.

## Graphical abstract

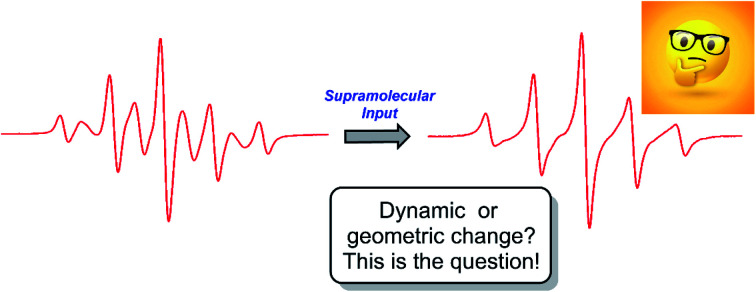

## Introduction

Interlocked molecules, such as rotaxanes and catenanes,^1^ have physical and chemical properties that are distinct from their individual covalent components.^2^ This consequence of the mechanical bond can be used to generate molecules with unusual reactivity^3^ or desirable behaviours such as the stabilisation of luminophores^4^ and organic conductors,^5^ caging of biomolecules,^6^ catalysts where the mechanical bond plays a key role,^7^ hosts for small molecules and ions^8^ and mechanically chiral structures from achiral building blocks.^9^ Interlocked molecules have also been used as platforms to study physical and chemical processes such as photo-induced through space transfer of electrons/electronic energy between substituents of the different covalent components;^10^ the mechanical bond ensures that the groups of interest cannot separate without the need for a direct covalent link that could confound the results.

The latter application of the mechanical bond relies on the restricted co-conformational motion of the mechanically bonded components, perhaps the most well-known feature of interlocked molecules given its role in the development of molecular machines.^11^ However, the mechanical bond also alters the conformational behaviour of the covalent subcomponents – most obviously the macrocyclic component of a rotaxane cannot adopt conformations in which the cavity is smaller than the axle it encircles! This suggests that interlocked molecules could be used to study properties and effects that depend on conformational behaviour without the need to covalently modify the structure of interest, although we are not aware of previous studies that have made use of this effect.

Biradical spin probes,^12^ and particularly those based on organic nitroxides,^13^ have attracted many investigators because of their potential usefulness in detecting changes in the distance between different molecules/regions of the same molecule in systems perturbed by an external stimulus. This can be performed both by measuring the dipole–dipole interactions between the two paramagnetic centres in frozen solution‡‡In non-viscous liquid solution, through space dipolar coupling is negligible due to rapid molecular tumbling and so only the electron exchange interaction is significant (see ref. 12). (by pulsed EPR techniques like PELDOR, DEER and RIDE spectroscopy)^14^ or by detecting through space spin exchange interactions,^15^ the strength of which, characterised by *J*, depends exponentially on the inter-radical distance and so can vary by several orders of magnitude.^12,16^ In the latter case, however, the observed EPR spectra of through-space spin exchanging biradicals depend also on the rate of exchange between states with different inter-radical distances (*i.e.* spectral density), as demonstrated theoretically by Luckhurst over 50 years ago.^17,18^ This is further complicated by the potential for through bond spin exchange between unpaired electrons when the radical centres are covalently linked.^12^

Thus, experimentally observed variations in the shape of the EPR spectra of nitroxide polyradicals cannot be related simply to changes in the value of *J* and thus to a variation in the distance of the radical centres; simulations based on Luckhurst's model§§For a biradical in solution in which each electron interacts equally with one nitrogen nucleus and the exchange integral *J* is modulated between two states “a” and “b” characterized by *J*_a_ and *J*_b_, respectively, the line width of the exchanging lines is given by: *T*_2_^−1^ = *a*_N_^2^*k*(*J̄*)/(2*J̄*)^2^ where *k*(*J̄*) is the spectral density 
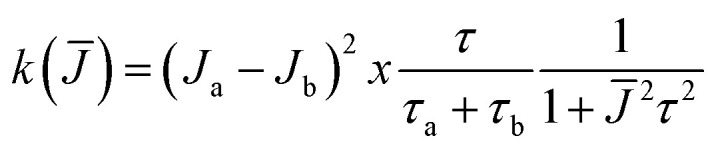
, *τ*_a_ and *τ*_b_ are the lifetimes of the two states and *τ* = *τ*_a_*τ*_b_/(*τ*_a_ + *τ*_b_).^15^ demonstrate^19^ that the qualitative form of EPR spectra may be the same for different combinations of *J* and rates of exchange between states with different values of *J*, and thus inspection of spectra is often insufficient to distinguish the contribution of these two effects. Indeed, separating the relative weight of these two effects can be challenging even in “simple” cases where the paramagnetic centres are covalently linked as any change in the structure of the polyradical will affect both the electronic properties (*i.e.* through-bond contributions to *J*) and molecular geometry (*i.e.* through-space contributions to *J*). Thus, the experimental determination of the contribution of these two effects by comparing the EPR spectra of model compounds is not a simple task, which can easily lead to incorrect conclusions.^18,20^

In principle, threading a linear axle component bearing radical units on its termini through a macrocycle to form a rotaxane should change its conformational properties (influencing through space interactions), without significantly altering the electronic properties of the system (through bond exchange). Here we demonstrate that this hypothesis is indeed correct; by studying biradical rotaxanes in comparison to their non-interlocked axles we show in solution that the qualitative form of the EPR spectra may be the same for different combinations of *J* and rate of conformations interconversion. Not only do our results validate the theoretical model of Luckhurst,^17,18^ they strongly reinforce the requirement that great attention must be paid when interpreting changes in the spectra of biradicals in response to chemical stimuli.

## Results and discussion

Although α-CD-, cyclophane-, octagonal metal ring- and crown ether-based rotaxanes containing biradicals have been described previously,^21^ in all cases their axles were characterized by a large degree of conformational freedom, making the detailed analysis of their EPR spectra extremely challenging. For this reason, we decided to prepare a family of rotaxane biradicals with well-defined conformational properties based on well-characterized units with relatively few rotatable bonds.^22^ To achieve this we used the active template^23^ Cu-mediated alkyne–azide cycloaddition (AT-CuAAC) approach, introduced in 2006 by Leigh and co-workers^24^ for the synthesis of rotaxanes, catenanes and knots and further developed by Goldup and co-workers to allow the synthesis of sterically constrained systems.^25^

**Scheme 1:**
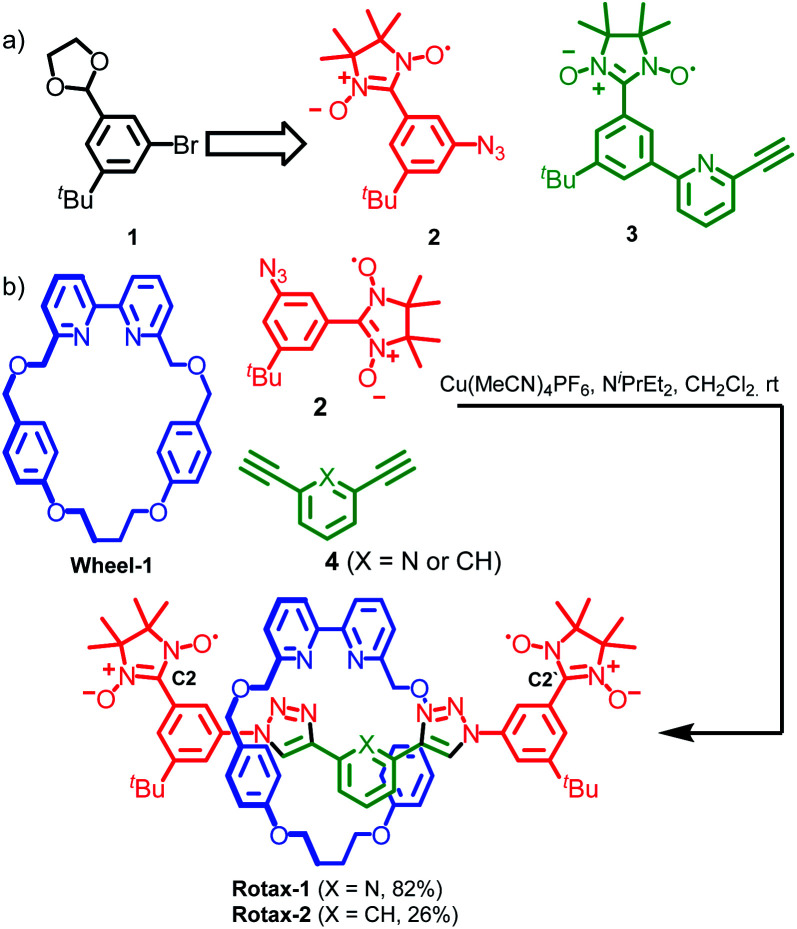
(a) Protected 3-bromo-5-^*t*^Bu-benzaldehyde starting material used in the synthesis of nitronyl nitroxide based half-axles 2 and 3 (used in the synthesis of Rotax-3, *vide infra*). (b) Synthesis of spin-labelled rotaxanes Rotax-1 and Rotax-2. The structures of corresponding axles, Axle-1 and Axle-2 are identical with the omission of the macrocycle.

Nitronyl nitroxides radicals^26^ were employed as paramagnetic substituents of the bulky stopper units because of (i) their stability under the conditions employed in the synthesis of the rotaxanes; (ii) their steric bulk which allows them to retain the small bipyridine macrocycles^27^ used in Goldup's AT-CuAAC approach when incorporated onto a 1,3,5-trisubstituted aromatic ring. Furthermore, nitronyl nitroxide-based half-axles, such as azide 2 and alkyne 3 (used in the synthesis of rotaxane 3, *vide infra*) suitable for the AT-CuAAC synthesis of rotaxanes are readily available from protected 3-bromo-5-(*tert*-butyl)-benzaldehyde 1 using standard procedures (Scheme 1a and ESI†). Accordingly, rotaxanes Rotax-1 and Rotax-2, which differ in the central aromatic unit of the axle, were synthesized from spin-labelled azide 2 and the corresponding bis-alkyne 4 in the presence of readily available macrocycle Wheel-1 (Scheme 1b). The corresponding non-interlocked axles, Axle-1 and Axle-2, which lack the encircling macrocycle, were also prepared for comparison.

### EPR features of bis(nitronyl nitroxides)

**Fig. 1:**
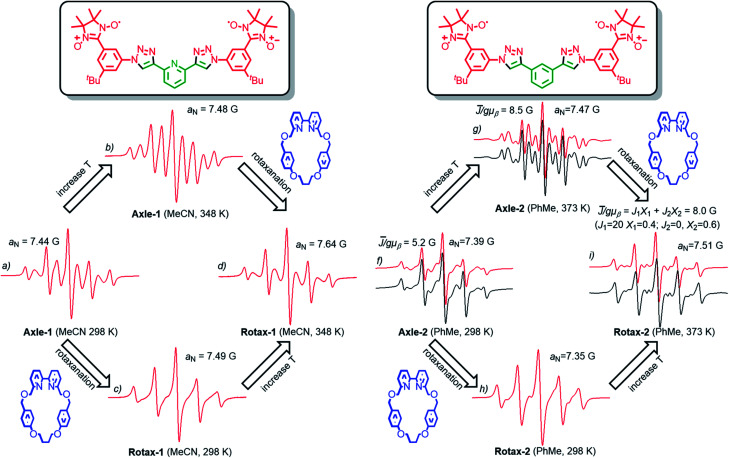
EPR spectra (in red) of Axle-1 (a–b), Rotax-1 (c–d), Axle-2 (f–g) and Rotax-2 (h–i).

The two nitrogen atoms (^14^N, *I* = 1) of a mono nitronyl nitroxide are magnetically equivalent and the coupling of the unpaired electron with them (measured by the isotropic hyperfine coupling constant *a*_N_) results in a five-line EPR spectrum of relative intensity of 1 : 2 : 3 : 2 : 1. When two chemically equivalent nitronyl nitroxides are magnetically coupled through an exchange interaction, the diradical gives an exchange-coupled EPR spectrum whose shape is dictated by the strength of coupling between the two electron spins, measured by the exchange integral, *J*, and the rate of exchange between states with different values of *J*. Considering the simple case (Fig. S50†), when *J* is negligible, the bis(nitronyl nitroxide) behaves as two independent monoradical nitroxides and the EPR spectrum is characterized by five hyperfine lines (see Fig. 1a). If *J* ≫ *a*_N_ (strong exchange), the spectrum is characterized by a nine-line pattern of relative integration 1 : 4 : 10 : 16 : 19 : 16 : 10 : 4 : 1 separated by *a*_N_/2 and analysis of the splitting pattern does not allow the direct determination of *J*. In intermediate situations (*J*/*gμ*_b_ ≈ *a*_N_), new hyperfine lines appear and the spectrum contains complex multiplets whose positions and intensities depend on the ratio *J*/*a*_N_ (see Fig. 1b). In this case the magnitude of *J* can be measured precisely by analysing the splitting pattern of the spectrum.^12^

When a biradical exists in several conformations having different values of *J* and their interconversion is fast, an averaged value of the exchange integral, *J̄*, would be observed in the corresponding EPR spectrum. In same cases EPR spectra are also characterized by considerable line width alternation, which is related to the modulation of *J* between different values;^17–20^ the widths of the five lines corresponding to the transition of the isolated monoradical are unaffected since their positions are independent of the modulation, but all other lines resulting from spin exchange are broadened since their positions depend on *J*.

### Predicted conformational behaviour of Axle-1 and Axle-2

In Axle-1 and Axle-2 and the corresponding rotaxanes, the contributions of through-bond coupling is expected to be negligible owing to the weak delocalization of the unpaired spin onto the phenyl fragment usually observed in nitronyl nitroxides,^28^ combined with the 1,3-substitution of the aromatic rings which prevents effective conjugation.^29^

**Scheme 2:**
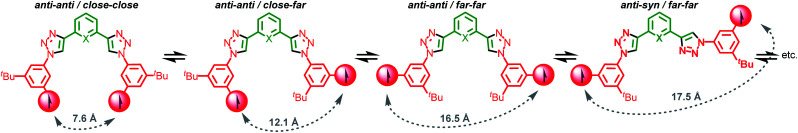
Limiting main conformations of the bis(triazolyl)pyridines (X = N) and bis(triazolyl)phenyl (X = CH) axles. Calculated distances shown here refer to Axle-2 (X = CH).

Thus, we can assume spin–spin coupling interactions in Axle-1, Axle-2 and the corresponding rotaxanes are dominated by through-space effects. If we consider the rotation of the bonds connecting the central aromatic ring and the two triazoles units and the bonds connecting the triazole rings to the aromatic ring bearing the paramagnetic unit, 9 limiting conformations can be predicted and, by considering the orientation of the triazole moieties with respect to the central aromatic unit (*syn* or *anti*) and the position of the nitroxide unit relative to the triazole C–H (“*close*” or “*far*”), these can be categorised into sets (Scheme 2). Stochastic dynamics (SD) simulations of Axle-2 in the gas-phase at 298 K using the AMBER*^30^ force field (see ESI†) allowed the average distances, 〈*r*〉, between C2–C2′ to be estimated for each conformation (Table S1†).

Inspection of these data shows that all *syn*–*syn* and *syn*–*anti* conformations are characterized by 〈*r*〉 > 13.7 Å, whereas in the *anti*–*anti* conformations, 〈*r*〉 values of 16.5, 12.1 and 7.6 Å were predicted for the *far*–*far*, *close*–*far* and *close*–*close* conformations respectively. SD analysis of Axle-1 revealed similar results. As the value of *J* tends to be small when two N–O bonds interacting through space are separated by a distance larger than 10–12 Å,^21*a*,31^ we can qualitatively predict that the exchange interaction between radical centres is negligible in all *syn* conformations and the *anti*–*anti*/*far*–*far* conformer, appreciable in the *anti*–*anti*/*close*–*far* conformer and strong in the *anti*–*anti*/*close*–*close* geometry.

Finally, the conformational behaviour of the 2,6-bis(1,2,3-triazol-4-yl)pyridine unit of Axle-1 has been thoroughly investigated by Hecht and co-workers^22^ and is known to predominantly adopt an *anti*–*anti* (*a*–*a*) conformation (Scheme 2) to avoid repulsive interactions between pyridine and triazole N lone pairs with a significant stabilization by 6.8 kcal mol^−1^ of the *anti* over the *syn* conformer.^22^ This conformational biasing is not present in Axle-2.

Based on this conformational analysis, both axles are expected to display strong exchange coupling in only one of the available conformations, the *anti*–*anti*/*close*–*close* geometry. Furthermore, as Axle-1 largely adopts one of the three *anti*–*anti* conformations, whereas Axle-2 is expected to exist as a complex mixture, on first inspection, the value of 〈*J*〉 is expected to be larger for Axle-1 than Axle-2. The same analysis applies to the corresponding rotaxanes.

### Comparison of the EPR spectra of interlocked and non-interlocked bis(nitronyl nitroxides)

The EPR spectra of Rotax-1/2 and Axle-1/2 were recorded in MeCN and PhMe at several temperatures and the key observations are highlighted here. The EPR spectrum of Axle-1 recorded at 298 K in MeCN (Fig. 1a) is characterized by 9 lines with evident line width alternation, implying a large average spin–spin interaction between the two radical units (*J̄* ≫ *a*_N_) with modulation of the line width due to exchange between conformations having distinct *J* values. As would be expected, the line width alternation was reduced at higher temperature where conformational exchange is more rapid; we observed a nine line spectrum at 348 K in which the relative intensities are very close to those expected when each of the unpaired electrons is coupled with four equivalent ^14^N atoms (see Fig. 1b). In contrast, in MeCN at room temperature the spectrum of Rotax-1 consists of five lines (Fig. 1c), while at higher temperatures (348 K, Fig. 1d) it comprises nine lines with strong line width alternation. At first glance, the variations in the spectral shape observed in MeCN at the same temperature between Axle-1 and Rotax-1 could be simply related to a decrease in the value of *J̄* in the interlocked structure compared to the non-interlocked axle. However, Luckhurst's model^18^ predicts that the spectral shape depends not only on the value of *J̄* but also on the rate of jumping between the different conformations.§ Furthermore, as in the case of Axle-1 and Rotax-1, it is not possible to distinguish between the two possible causes of the observed change if *J̄* cannot be directly measured.

Thus, the mechanical bond could affect one or both of *J̄* and the rate of conformational exchange and it is not possible to ascribe the line-width differences simply to variations of *J̄* alone. Conformations in which the two nitroxide units of Axle-2 are far apart (Scheme 2) are expected to be significantly more populated (*vide supra*) and thus a decrease of *J̄* should be expected for Axle-2 relative to Axle-1. Indeed, in PhMe both at 298 K and 373 K (Fig. 1f and g, red lines), the EPR spectra of Axle-2 were characterized by a complex pattern as expected when *J̄*/*gμ*_b_ ≈ *a*_N_. Although theoretical simulations perfectly reproduced the position of spectral lines, their intensity was not correctly replicated because of the significant alternating linewidths. Thus, to properly reproduce the spectra of Axle-2, each line was allowed to vary in width by maintaining the position and the intensity predicted theoretically for a given value of the exchange integral (see ESI† for details).

Using this approach, we were able to simulate the spectra and to obtain a value of *J̄*/*gμ*_b_ = 5.2 G at 298 K and *J̄*/*gμ*_b_ = 8.5 G at 373 K for Axle-2 (see black lines in Fig. 1f and g). Since the value of *J̄* does not change significantly with temperature, it appears that the narrowing of spectral lines with increasing *T* can be attributed to a higher rate of conformational interconversion. In contrast, the EPR spectrum of Rotax-2 at 373 K (Fig. 1i) was accurately modelled as the superimposition two groups of conformers, for one of which *J* = 0 (∼60% of the total signal) and the other with *J*/*gμ*_b_ = 21 G (40%), resulting in an average value *J̄*/*gμ*_b_ = 8.0 G.¶¶Because of severe line broadening, determination of the two spectral components was instead not possible at 298 K (Fig. 1h). The observation of distinct signals for two groups of conformers of Rotax-2 implies that the transition between them is slow on the EPR time scale.

Given that value of *J̄* value measured in PhMe at 373 K for Rotax-2 (8.0 G) is very close to the value found for Axle-2 (8.5 G) at the same temperature, it appears that the encircling macrocycle does not directly affect the value of *J̄* by perturbing the conformational equilibrium but rather that the major effect of the encircling macrocycle is to slow the rate of conformational interconversion. If this interpretation is extended to the Axle-1/Rotax-1 couple, variations in the spectral shape observed at the same temperature in MeCN (Fig. 1a and c) are proposed to be due to, and are consistent with, a slower rate of jumping between the different conformations in the interlocked structure, rather than a decrease in the *J̄* value, as could be concluded simply on the basis of the five line spectrum observed with Rotax-1 at 298 K.

**Fig. 2:**
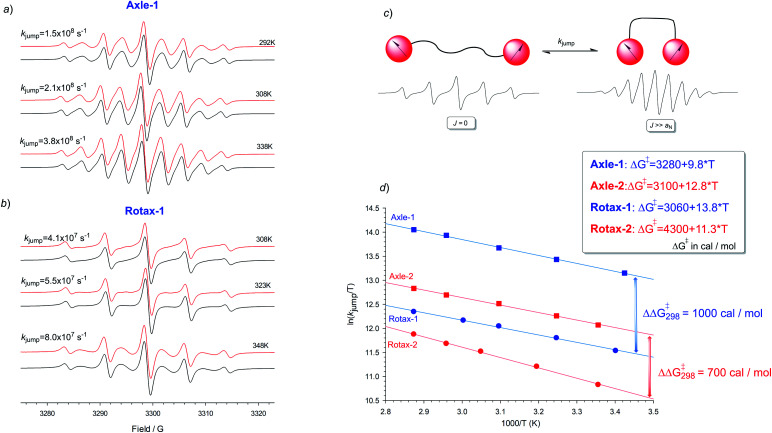
EPR spectra of Axle-1 (a) and Rotax-1 (b) recorded in MeCN at various temperatures. In black are reported the corresponding theoretical simulations obtained by using the rates of exchange reported in the figure and by assuming a two-state jump model (c) where *k*_jump_ represents the apparent rate of jumping between two set of conformations having and *J* = 0 and *J* ≫ *a*_N_. (d) Plots of ln(*k*_jump_) *versus* 1000/*T* for the different biradicals.

This interpretation is supported by kinetic analysis based on a simple two-state jump model (see Fig. 2c)^17^ and simulating the exchange broadened EPR spectra by using density matrix theory which covers the full ranges of rates. In this simplified model only two extreme conformations, one in which *J* ≫ *a*_N_ (strongly interacting) and in which *J* = 0 (non-interacting), are considered and an apparent rate of exchange, *k*_jump_, between these conformations which modulates the exchange integral, produces an alternating linewidth which was determined as an empirical parameter of Axle-1, Axle-2, Rotax-1 and Rotax-2. In all cases the simulations obtained with this approximate two state model are in excellent agreement with the experimental spectra (see Fig. 2a and b for Axle-1 and Rotax-1 in MeCN, see ESI† for equivalent data for Axle-2 and Rotax-2). The validity of this simple model is supported by the good logarithmic dependence of *k*_jump_*vs.* 1/*T* (Fig. 2d), consistent with a single operative mechanism determining line width alternation.

Comparison between the jump rates measured in the pairs Axle-1/Rotax-1 or Axle-2/Rotax-2, afforded an apparent free energy increase for exchange between the strongly and weakly interacting conformations due to the encircling macrocycle as ΔΔ*G*^‡^ = 1.0 and 0.7 kcal mol^−1^, respectively (Fig. 2). These data definitively confirmed that the encircling macrocycle has a significant effect in reducing the rate of interconversion between the various conformations (*close*–*close*, *close*–*far* and *far*–*far*).

The important conclusion from the above analysis is that very similar EPR spectral variations can clearly be demonstrated to arise due to two different effects. The EPR spectra at 298 K of Axle-1 (MeCN) and Axle-2 (PhMe) contain 9 lines (with significant line width alternation) and 5 lines respectively and this effect is attributed to the higher value of 〈*J*〉 in the former case due to a preference for the *anti*–*anti* family of conformations. Conversely, the same observed difference between the EPR spectra of Axle-1 and Rotax-1 (5 lines at 298 K in MeCN) is due to slower exchange (*i.e.* reduced *k*_jump_) between conformations because of the mechanical bond without perturbation of the conformational equilibrium (*i.e.* little or no change in *J̄*).

### A case study: Rotax-3

**Fig. 3:**
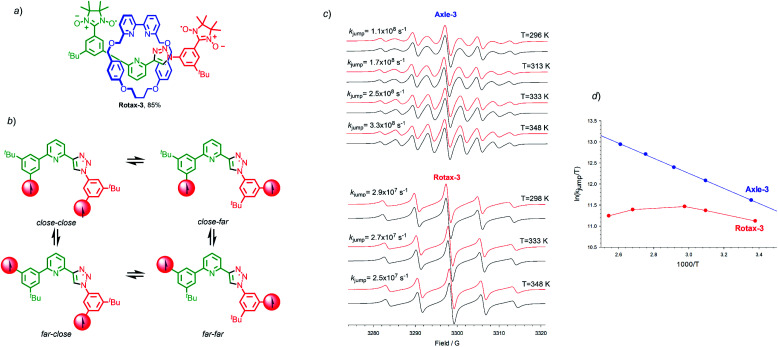
(a) Structure of Rotax-3. The structures of corresponding axle, Axle-3 is identical with the omission of the macrocycle. (b) Main conformations that can be depicted for Axle-3. (c) EPR spectra of Axle-3 and Rotax-3 recorded in MeCN at various temperatures. In black are reported the corresponding theoretical simulations obtained by using the rates of exchange reported in the figure and by assuming a two-state jump model where *k*_jump_ represents the apparent rate of jumping between two set of conformations having and *J* = 0 and *J* ≫ *a*_N_ (see text). (d) Plots of ln(*k*_jump_) *versus* 1000/*T* for the apparent rate of jumping between two set of conformations having and *J* = 0 and *J* ≫ *a*_N_, for Axle-3 (blue symbols) and Rotax-3 (red symbols) in *tert*-butylbenzene.

Finally, we examined the EPR properties of Axle-3 and Rotax-3 (Fig. 3a), which contain one fewer aromatic ring between the paramagnetic centres than Axle-1 and were synthesised in an equivalent manner to Rotax-1/2 from spin-labelled azide 2 and spin-labelled alkyne 3 (see Scheme 1 and ESI†). In this case, four main conformations relative to the phenyl-triazole and phenyl-pyridine bonds can be expected (Fig. 3b) and averaged distances 〈*r*〉 between C2 and C2′ in the paramagnetic heterocycle units were estimated by SD (Table S1†). Inspection of these data shows that despite lacking a second triazole ring, the values of 〈*r*〉 are very similar to those estimated for the main conformations of Axle-1. Thus, it can be conceptually envisaged that in Axle-3 the spin–spin exchange interaction should be comparable to that of Axle-1.

The EPR spectrum of Axle-3 (Fig. 3c), recorded in MeCN at 296 K was characterized by the presence of 9 lines (*a*_N_ = 7.53 G, *J̄* ≫ *a*_N_),||||Although with Axle-3 the two nitroxides are no longer equivalent and this could affect spectral shape, it has been reported (see ref. 26) that differences in *a*_N_ values are less than 0.2 G, that is much smaller than the intrinsic line width, even when substituents at the phenyl ring vary from strongly electron donating (*e.g.* –NMe_2_) to strongly electron withdrawing (*e.g.* –NO_2_). Thus, we are confident that in our case the difference will be even smaller and so can be safely neglected. similar to that observed for Axle-1. Similarly, the spectra of Axle-3 show an obvious line width alternation which was significantly reduced at 348 K where the relative intensities of the nine lines are close to those expected when each of the unpaired electrons is coupled with four equivalent ^14^N atoms (Fig. 3c). Changing the solvent to ^*t*^Bu-benzene did not alter the appearance of the spectrum obtained (see ESI†). In contrast, Rotax-3, the interlocked analogue of Axle-3, gives rise to five line spectra both in MeCN (*a*_N_ = 7.52 G, Fig. 3c) and *tert*-butylbenzene (*a*_N_ = 7.41 G). However, in this case increasing the temperature does not significantly change the EPR spectral shape in either solvent (Fig. 3c). Although, the rate of exchange in Rotax-3 is close to the lower limit of the EPR time scale, modulation of spin exchange is still evident as the intensities of the spectral lines do not fit with the expected 1 : 2 : 3 : 2 : 1 ratio (Fig. S39†).

Simulated EPR spectra of Axle-3 (Fig. 3c), were used to determine *k*_jump_ for exchange between two set of conformers having *J* = 0 and *J* ≫ *a*_N_, at different temperatures and the logarithmic dependence of *k*_jump_ as a function of temperature (Fig. 3d). As previously observed with Axle-1 and Axle-2, an excellent linear dependence was found in this case both in MeCN and *tert*-butylbenzene, suggesting that the variation in spectral shape with temperature results from the increased rate of conformational exchange. As with Rotax-1/2, the encircling macrocycle results in lower values of *k*_jump_. Conversely, in the case of Rotax-3 the logarithmic dependence of *k*_jump_*vs.* 1/*T* is non-linear both in MeCN and *tert*-butylbenzene (Fig. 3d).

Thus, it appears that varying the temperature has a significant effect not only on the rate of jumping, but also on the value of *J̄*, suggesting that the conformational equilibrium is also perturbed. Based on this analysis, it appears that the shorter axle of Rotax-3 leads to an increase in the importance of steric factors; the steric hindrance around the bonds connecting the aromatic ring encircled by the macrocycle leads to conformer distributions, and thus to *J̄* values, more sensitive to temperature variations. Surprisingly however, the EPR spectra obtained at 298 K and 393 K are extremely similar, suggesting that these two effects tend to counterbalance each other leading to an overall small variation of spectral shape with temperature. Once again, apparently similar EPR spectra of biradicals show line width alternation which is the result of different relative weight of the rate of jumping and spin exchange.

## Conclusions

From a general point of view, nitroxide spin probe-based methodologies provide an important tool for the investigation of supramolecular assemblies and we expect that this technique will expand in this important domain of chemistry. Biradicals have a number of advantages over monoradicals as probes as, by measuring spin exchange interactions, it is possible to study the intramolecular mobility and three-dimensional structure of the biradical, and these are generally most sensitive to change in the state of the supramolecular assembly. The family of rotaxane biradicals investigated here proved particularly useful for the interpretation of the electron resonance spectra of dynamic biradical systems in solution when the number of spectral lines may often be obscured by extreme line broadening and very similar spectra can be the result of different effects on line width alternation. We believe this work can significantly help in the interpretation of such spectra.

## Author contributions

L. G. performed the synthesis and characterised the compounds. P. F. measured EPR spectra. E. M. contributed to data analysis, and manuscript refinement. S. M. G. and M. L. designed the project, analyzed the results and wrote the manuscript.

## Conflicts of interest

There are no conflicts to declare.

## Supplementary Material

SC-012-D1SC01462E-s001
