# Supplementary material for: Spin-labelled mechanically interlocked molecules as models for the interpretation of biradical EPR spectra
Source: Chem Sci. 2021 May 14;12(24):8385–93. doi: 10.1039/d1sc01462e (PMC8221063; doi:10.1039/d1sc01462e)
Supplement: SC-012-D1SC01462E-s001 [file SC-012-D1SC01462E-s001.pdf]

Electronic Supplementary Material (ESI) for Chemical Science.
This journal is © The Royal Society of Chemistry 2021

Supporting Information for:

Spin-labelled Mechanically Interlocked Molecules as Models for the Interpretation of Biradical EPR Spectra

Lorenzo Gualandi, Paola Franchi, Elisabetta Mezzina, Stephen M. Goldup, Marco Lucarini

Materials and methods………………………………………….. S2
General synthetic aspects………………………………………….. S2
Preparation of compound **1** S4
Preparation and characterization of compounds **2b, 2c, 2**……… S6
Preparation and characterization of compounds **3a, 3b, 3c, 3**…. S12
Preparation and characterization of Rotaxanes **1-3** …………… S21
Preparation and characterization of Axles **1-3** ………………….. S25
EPR spectroscopy ………………………………………………. S30
Dynamic simulations ……………………………………………. S33
References …………………………………………………….. S34

Materials and methods

NMR analysis were recorded on Varian Mercury 400 MHz, Bruker AV400 and AV3-400 spectrometers at 298 K, using the solvent peaks as internal standards. Chemical shifts are reported in parts per million (ppm, δ scale).

ESI-MS Mass spectrometry analyses were performed on Waters ZMD 4000, ZQ 4000 and TQD mass spectrometers, the sample was prepared in methanol or acetonitrile. LC-MS analysis was performed with a Waters TQD mass spectrometer equipped with UHPLC injection with a BEH C18 column; MeCN/hexane gradient + 0.2% formic acid.

GC-MS was performed on an Agilent 7890 Gas Chromatographer paired with Agilent 5973 Mass selective detector, CH_2Cl_2 was used as solvent for the preparation of the samples.

UV-visible experiments were performed on a JASCO V-550 spectrometer.

Reactions were monitored by TLC chromatography and the formation of the products was visualized with UV, I_2 or ninhydrin. Column Chromatography was performed on Merck silica gel (230–400 mesh).

Elemental analysis of the nitroxide radicals were performed on a Thermo Flash 2000 CHNS/O analyzer.

All reagents and solvents were used as received, without further purification. When necessary solvents were degassed by bubbling nitrogen.

General synthetic aspects

3-bromo-5-*tert*-butyl-benzaldehyde,[1] 2,6-diethynylpyridine,[2] tosyl-azide[3] 2,3-bis-hydroxyl-amino-2,3-dimethylbutane (BHA),[4] 6-chloropyridyl-2-boronic acid[5] and macrocycle **Wheel-1**[6] were synthesized according to literature procedures.

Azide **2** was obtained starting from **1** that after lithiation with *n*-BuLi, successive reaction with tosylazide (TsN_3), and removal of the acetal protecting group gave the free aldehyde **2b** which was then condensed with 2,3-bis-hydroxylamino-2,3-dimethylbutane (BHA) in the presence of a catalytic amount of *para*-toluenesulfonic acid (TsOH) affording the radical precursor dihydroxyimidazolidine **2c**. Oxidation of **2c** with $NaIO_4$ in CH_2Cl_2/H_2O yielded azide functionalized spin-labelled half-axle **2** (Scheme 1S).

For the synthesis of the ethinyl(pyridyl) functionalized paramagnetic half-axle **3,** compound **1** was subjected to a palladium assisted Suzuki-Miyuara cross-coupling with 6-chloropyridyl-2-boronic acid to give compound **3a**. It was subsequently reacted with TMS-acetylene in DMF at 120°C to give the free aldehyde **3b** that was then subjected to the same synthetic protocol as **2**. Treatment of **3d** with K_2CO_3 deprotected the alkyne functionality to give compound **3** (Scheme 1S).

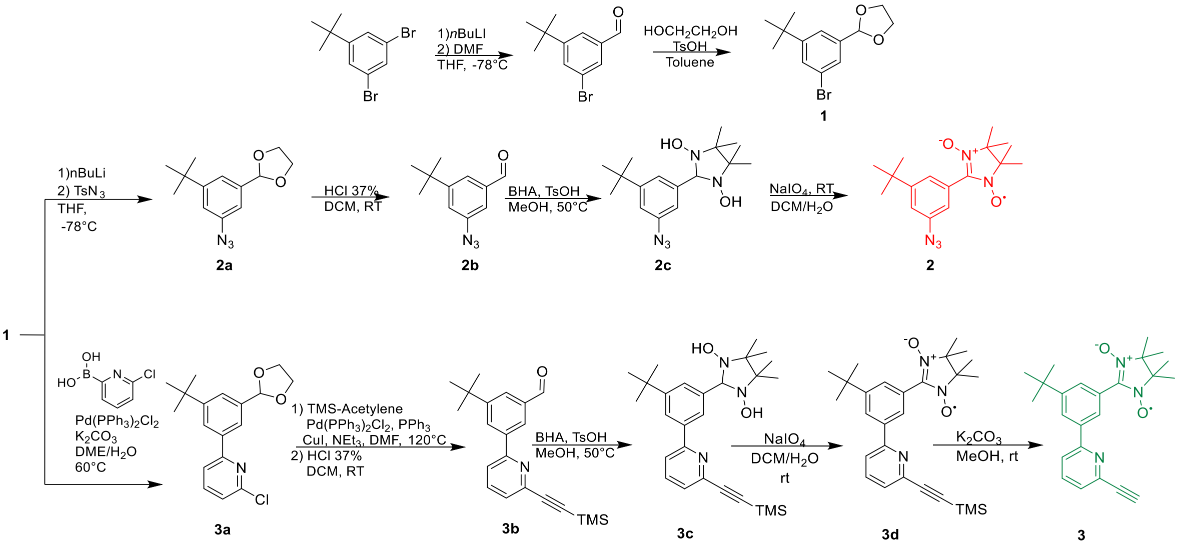

Scheme 1S. Synthesis of the spin-labelled half-axles.

The spin-labelled half-axles **2** and **3** were then used both in the presence or in the absence of the macrocycle **Wheel-1** for the synthesis of the target rotaxanes or non-interlocked axles, respectively. The radical units well tolerated AT-CuAAC conditions which employed copper(tetrakis-acetonitrile) hexafluorophosphate as catalyst for the click reaction. In general, the macrocycle **Wheel-1** (1eq) was mixed with and a sub-stochiometric amount of copper (0.96 eq) to form the Cu^{I}/**Wheel-1** complex before the addition of the azide and alkyne half-axles to the reaction vessel. The addition of DIPEA starts the reaction that leads to the target interlocked molecules. Once the reaction is complete, copper is removed upon treatment with a saturated ethylenediaminetetraacetic acid/ammonia 17.5 % solution (EDTA/NH_3). Because of the presence of paramagnetic centers NMR could not be used for the characterization of the final bis(nitronyl nitroxide)s and EPR, MS and UV-Vis were employed for this purpose.

The spin-labelled axles and rotaxanes were found to be fairly persistent. Actually, upon storage at 4°C for few months, the EPR analysis evidenced the presence of less than 10% of imino nitroxide derivatives, produced by decomposition of the nitronyl nitroxide diradical. To avoid the presence of EPR active imino nitroxide byproducts, the samples were always purified *via* column chromatography before EPR analysis.

Synthesis of 2-(3-bromo-5-(tert-butyl)phenyl)-1,3-dioxolane (1)

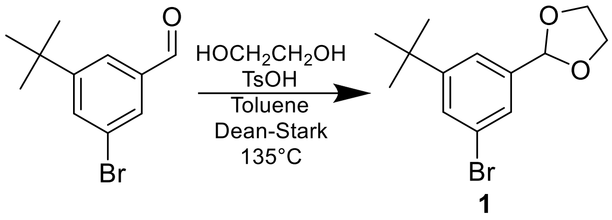

3-bromo-5-(tert-butyl)-benzaldehyde (4.37g, 18.15 mmol), ethylene glycol (5.06 mL, 90.78 mmol, 5 eq.), *para*-toluenesulfonic acid (TsOH, 311 mg, 10% mol) were placed in a round bottom flask and solubilized with toluene (125 mL). The solution was heated to reflux at 150 °C for 20 h in a Dean-Stark apparatus. The progression of the reaction was checked via TLC (eluent: petroleum ether/EtOAc 9:1). The reaction mixture was then cooled to RT and EtOAc (125 mL) was added. the solution was then washed with saturated $NaHCO_3$ (250 mL) and brine (250 mL), dried on $MgSO_4$, filtered, and the solvent removed *in vacuo*. The crude mixture was purified via column chromatography (SiO_2, eluent: petroleum ether/EtOAc 9:1) to yield compound **1** as a pale-yellow oil (4.85g, 93%). ^{1}H NMR (400 MHz, $CDCl_3$) δ = 7.50 (m, 1H), 7.46 (m, 1H), 7.40 (m, 1H), 5.76 (s, 1H), 4.13-3.03 (m, 4H), 1.31(s, 9H). ^{13}C NMR (101 MHz, $CDCl_3$) δ 153.79, 139.76, 129.62, 126.72, 122.52, 122.45, 103.22, 65.46, 35.08, 31.30. GC-MS (E.I., *m/z*, %): 283 [M-H$^+$, 95%].

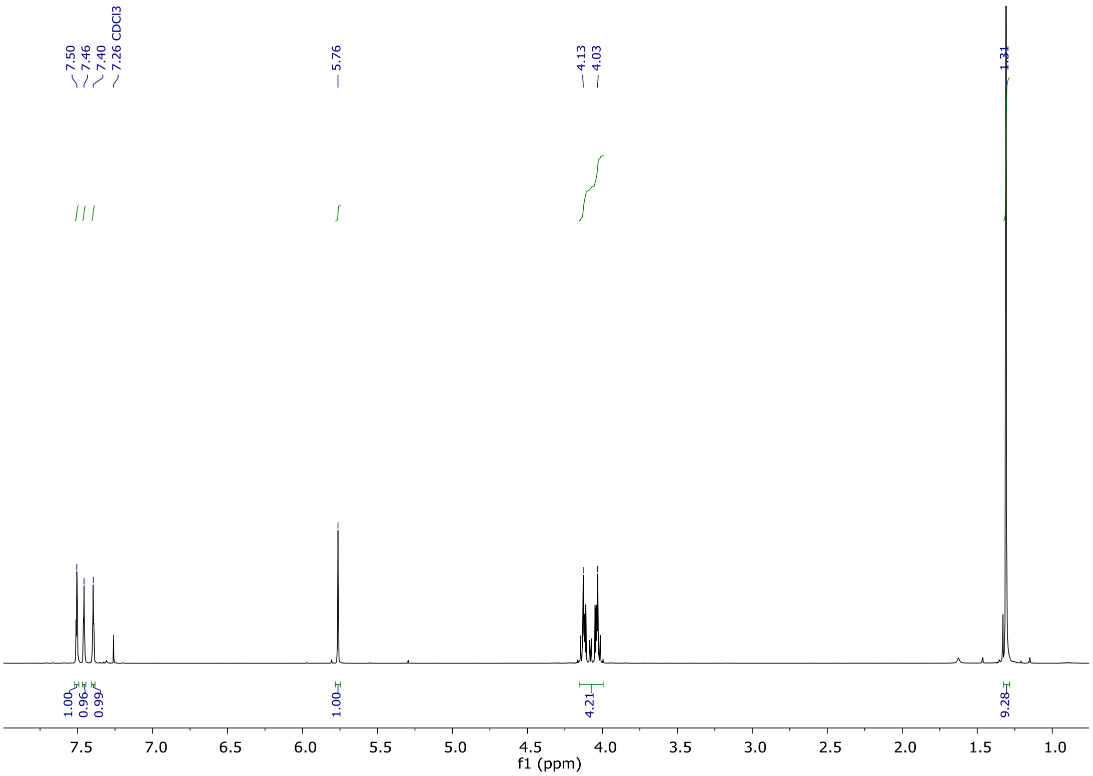

Figure S1. ^{1}H-NMR (400 MHz) of **1** in $CDCl_3$ at 298K.

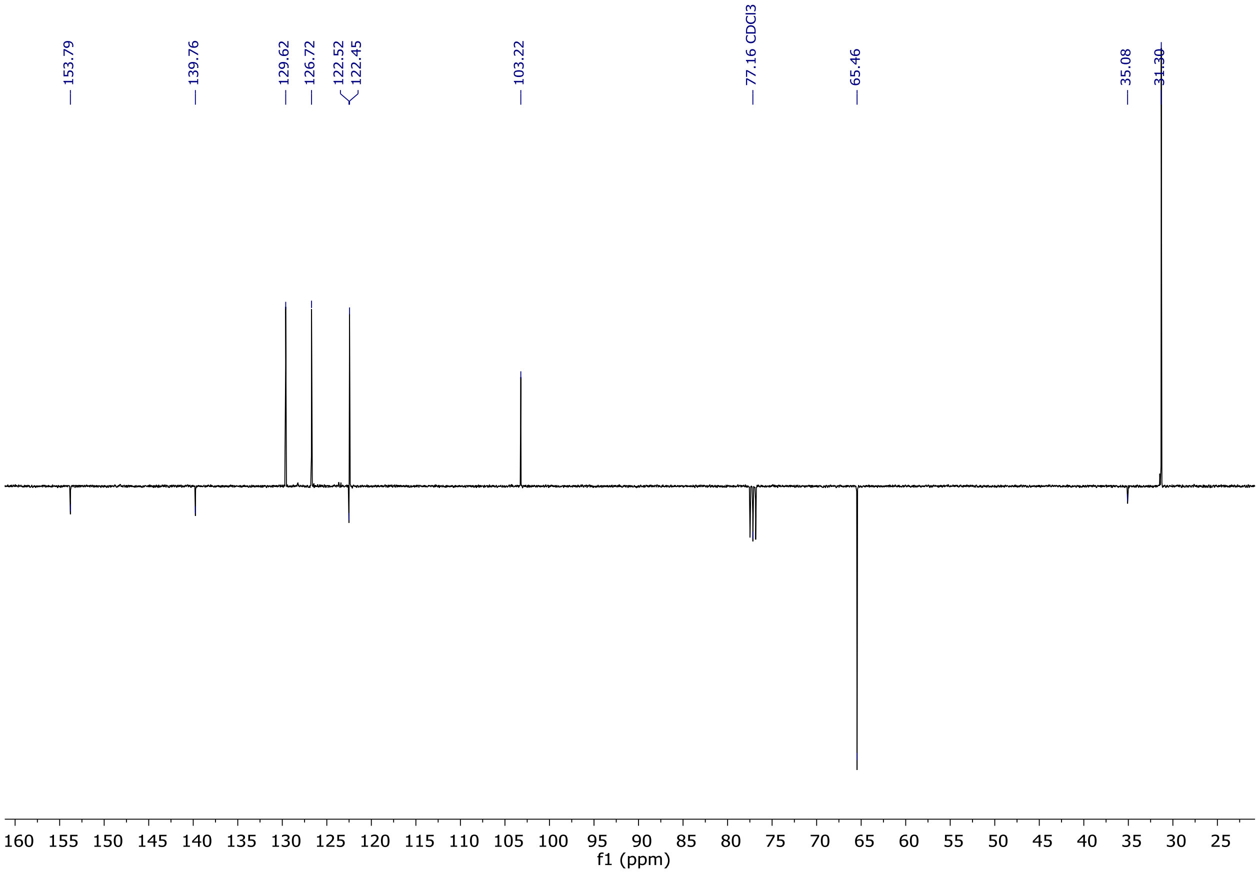

Figure S2. *J* MOD NMR (101 MHz) of **1** in $CDCl_3$ at 298K.

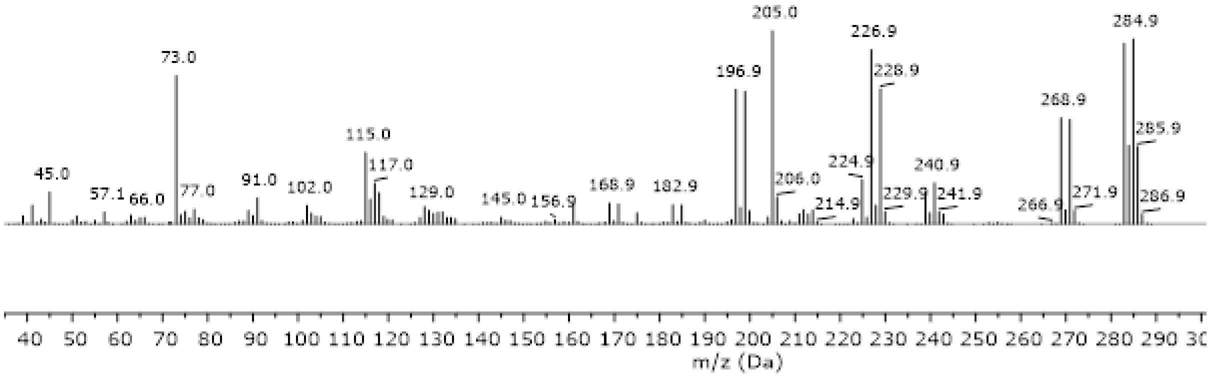

Figure S3. MS spectrum (EI) of compound **1.**

Synthesis of 3-Azido-5-(*tert*-butyl)benzaldehyde (2b)

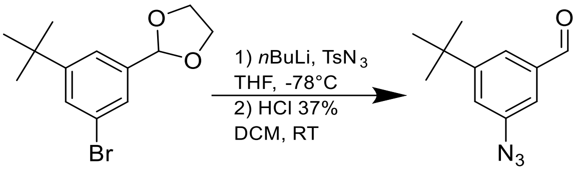

A solution of **1** (1.07 g, 3.74 mmol) in dry THF (100 mL) under N_2 was cooled to -78°C and treated dropwise with a solution of *n*-BuLi 2.5 M in hexane (1.72 mL, 4.30 mmol, 1.15 eq.) over 15 minutes. The solution was left to stir 30 minutes and then a solution of TsN_3 (1.10 g, 5.61 mmol, 1.5 eq.) in dry THF (14 mL) was added dropwise. The deep red solution was stirred at -78°C for 30 minutes and then warmed to RT and stirred for 16 h. The reaction was quenched with 100 mL of H_2O and the THF was removed *in vacuo*. The aqueous layer was then extracted with Et_2O (3 × 100 mL). The combined organic layer was the washed with water (300 mL) and then with brine (300 mL). The solution was dried on $MgSO_4$, filtered and concentrated in *vacuo* to yield **2a** as a yellowish oil. The crude mixture was used for the deprotection without further purification. (835 mg, 91%). ^{1}H NMR (400 MHz, Acetone-*d$_6$*) δ = 7.42 (m, 1H), 7.18 (m, 1H), 7.11 (m, 1H), 5.83 (s, 1H), 4.21-4.07 (m, 4H), 1.42 (s, 9H). The crude dioxolane protected aldehyde (500 mg, 2.05 mmol) was dissolved in CH_2Cl_2 (25 mL) and treated with HCl 37% (111 µL, 2.05 mmol, 1 eq.). The mixture was stirred overnight at RT. The progress of the reaction was monitored via TLC (eluent: petroleum ether/CH_2Cl_2 1:1). The mixture was neutralized with sat. $NaHCO_3$, the organic layer was then washed with H_2O (25 mL) and brine (25 mL) and dried on $MgSO_4$, filtered and the solvent removed *in vacuo*. Compound **2b** was purified with column chromatography (SiO_2, Eluent: petroleum ether 100% to petroleum ether/ CH_2Cl_2 1:1) to yield title compound **2b** as a yellowish oil (337 mg, 80%). ^{1}H NMR (400 MHz, CD_3CN) δ = 9.96 (s, 1H) 7.75(m, 1H), 7.38 (m, 1H), 7.33 (m, 1H), 1.34 (s, 9H). ^{13}C NMR (101 MHz, CD_3CN) δ = 193.02, 155.51, 142.04, 138.92, 124.69, 123.07, 118.26, 117.13, 35.77, 31.19.

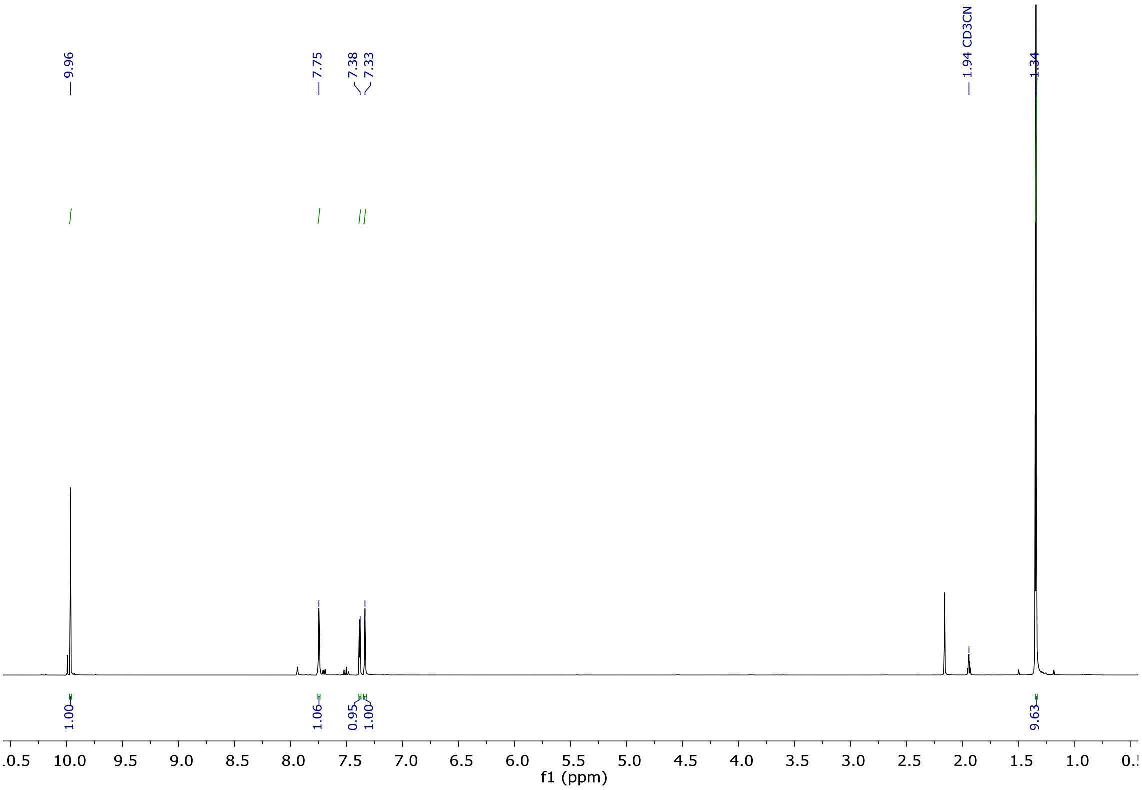

Figure S4. ^{1}H-NMR (400 MHz) of **2b** in CD_3CN at 298K.

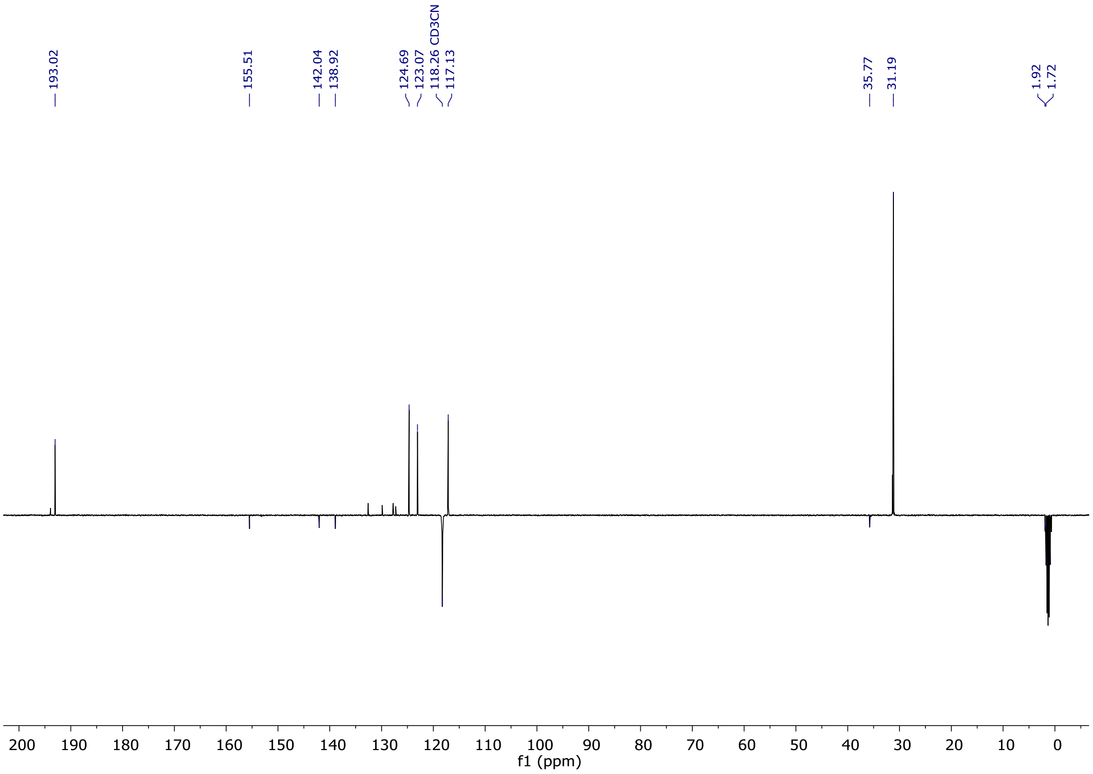

Figure S5. J MOD NMR (101 MHz) of **2b** in CD_3CN at 298K.

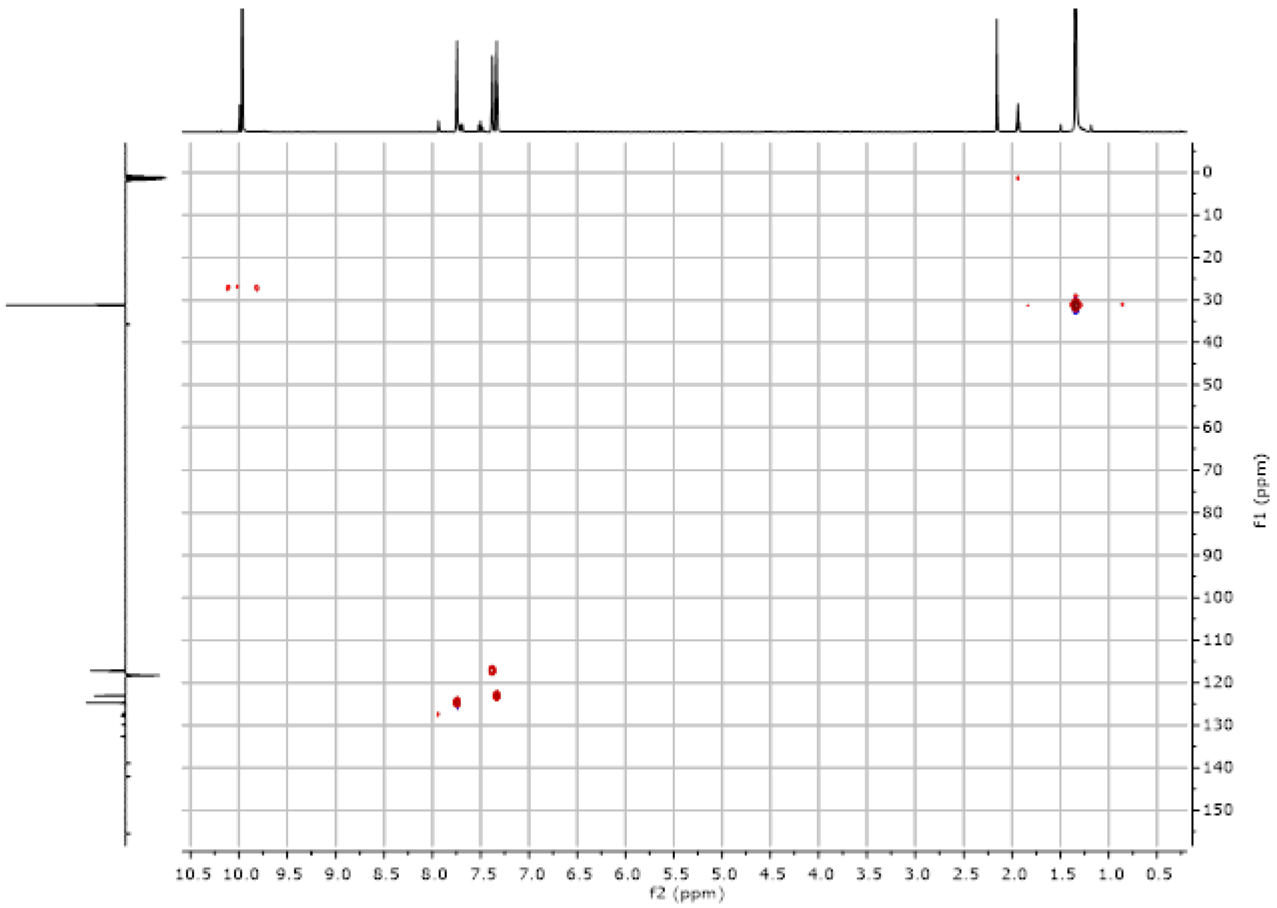

Figure S6. HSQC NMR of **2b** in CD_3CN at 298K.

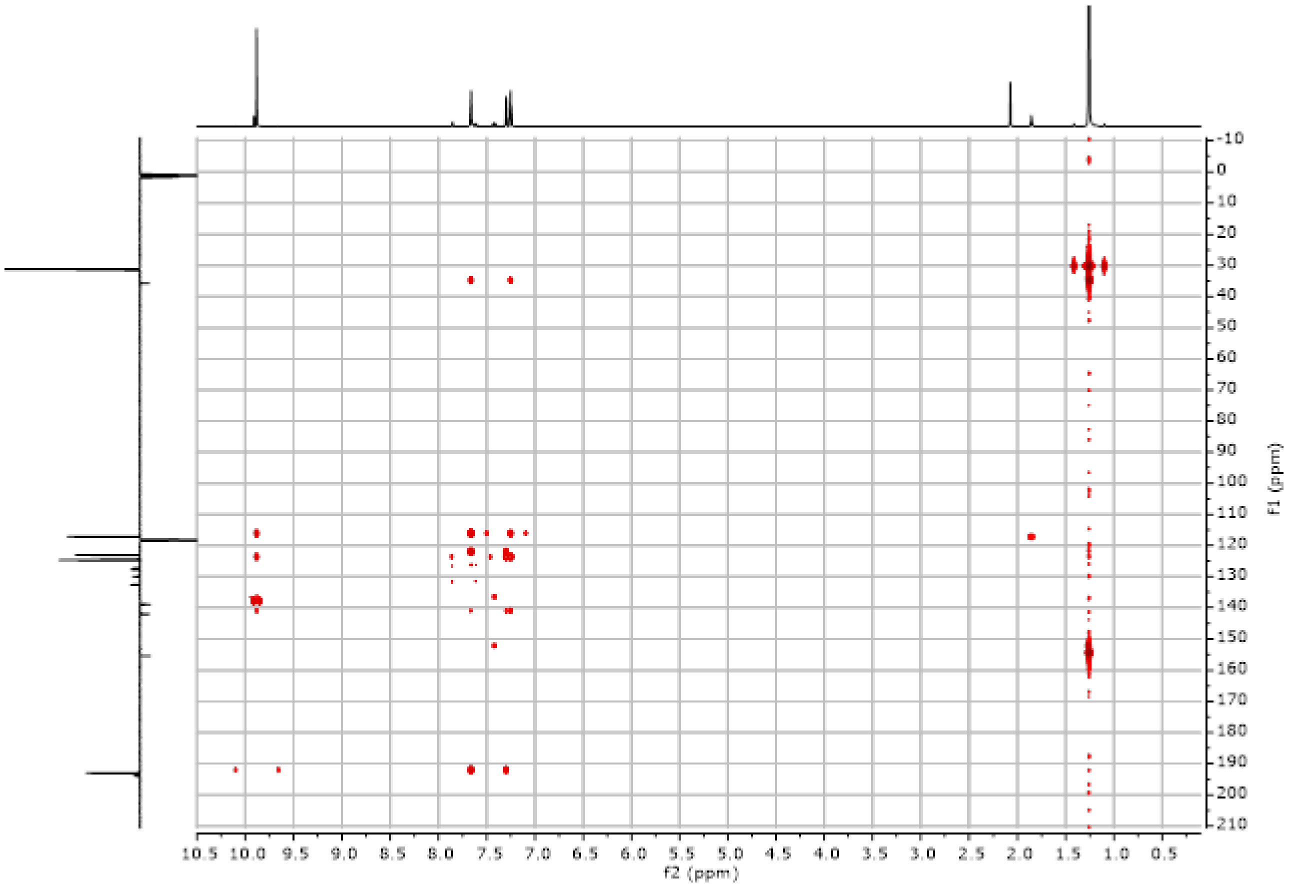

Figure S7. HMBC NMR of **2b** in CD_3CN.

Synthesis of 2-(3-azido-5-(*tert*-butyl)phenyl)-4,4,5,5-tetramethylimidazolidine-1,3-diol (2c)

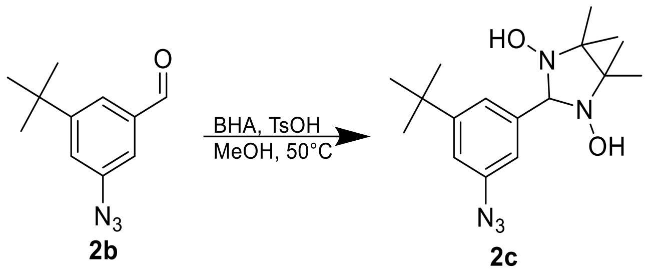

A degassed solution of aldehyde **2b** (337 mg, 1.65 mmol), 2,3-bis-hydroxylamino-2,3-dimethyl-butane (BHA, 244 mg, 1.65 mmol, 1 eq.), TsOH (15 mg, 0.082 mmol, 5 mol%) in dry MeOH (7 mL) was stirred under N_2 at 50 °C for 18 h. The cloudy reaction mixture was then cooled to RT, concentrated *in vacuo* to a minimal volume and the product precipitated with pentane, filtered and washed again with pentane to yield **2c** as a white powder (346 mg, 63%) that was used without further purification in the next step. ^{1}H-NMR (400 MHz, DMSO-d_6) δ = 7.84 (s, 2H), 7.33 (m, 1H), 7.08 (m, 1H), 6.95 (m, 1H), 4.51 (s, 1 H), 1.28 (s, 9H), 1.08-1.05 (ds, 12H). ^{13}C NMR (101 MHz, DMSO-d_6) δ = 152.17, 143.88, 138.30, 122.63, 115.71, 115.11, 90.16, 66.23, 39.52, 34.58, 31.30, 31.05, 24.36, 17.17. ESI-MS (+, *m/z*, %): 334.5 [M+H$^+$, 100].

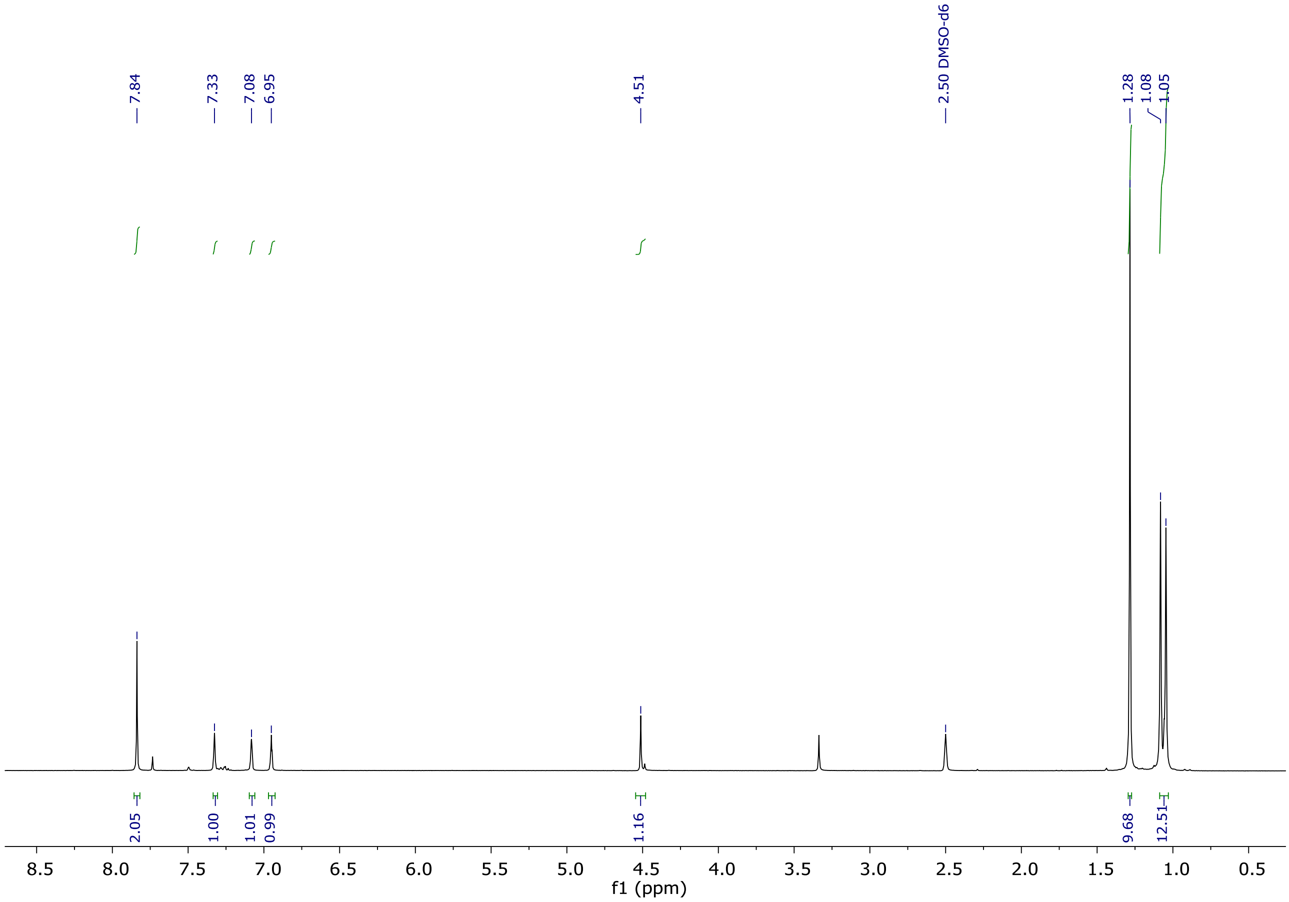

Figure S8 ^{1}H-NMR (400MHz) of **2c** in DMSO-d_6 at 298K.

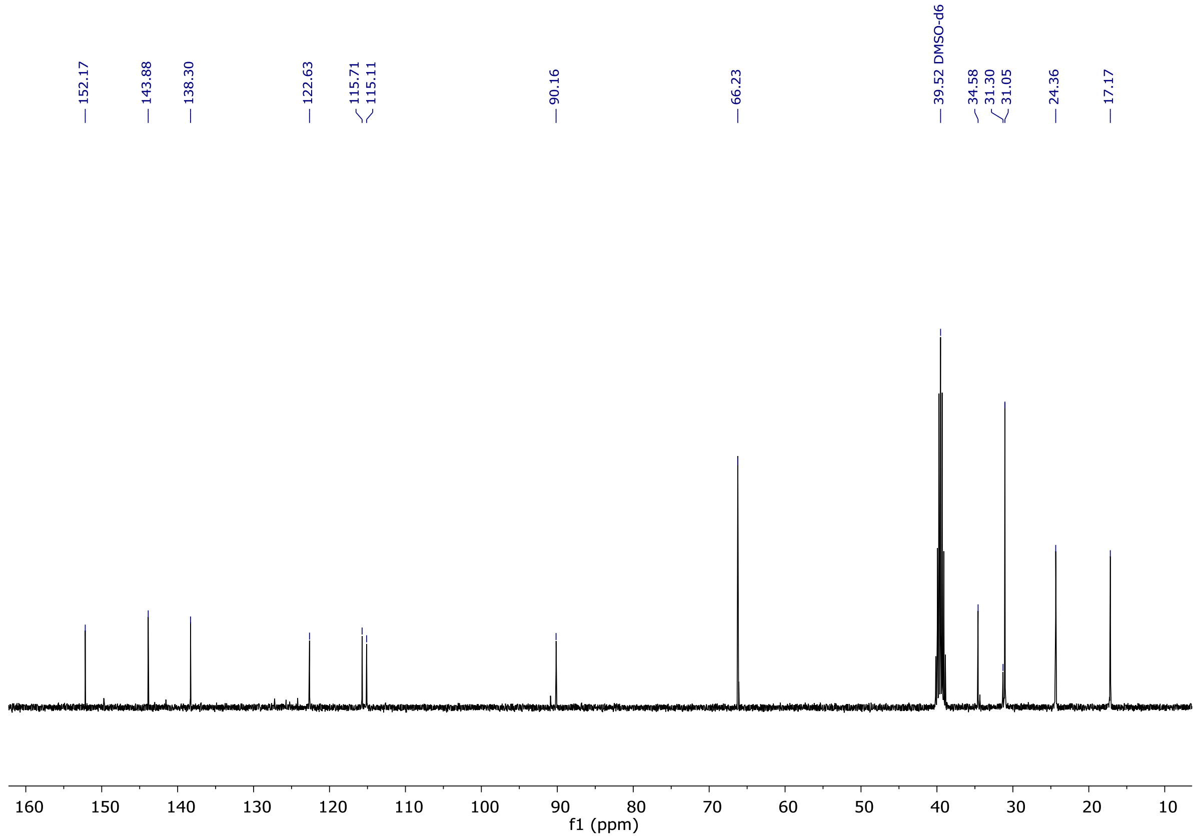

Figure S9 ^{13}C-NMR (101MHz) of **2c** in DMSO-d_6 at 298K.

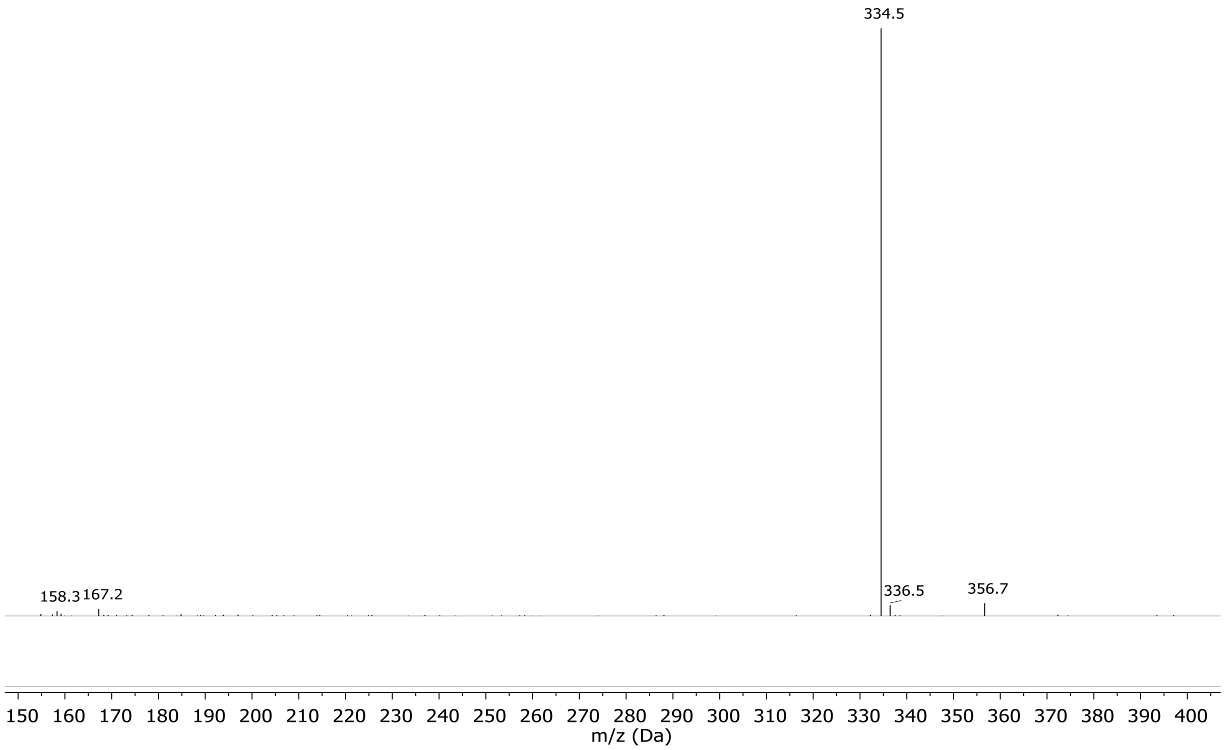

Figure S10. MS spectrum (ESI +) of compound **2c.**

Synthesis of 1-(4,4,5,5-tetramethyl-1-oxyl-3-oxide- imidazoline-2-yl)-3-azido-5-*tert*-buthylbenzene (2)

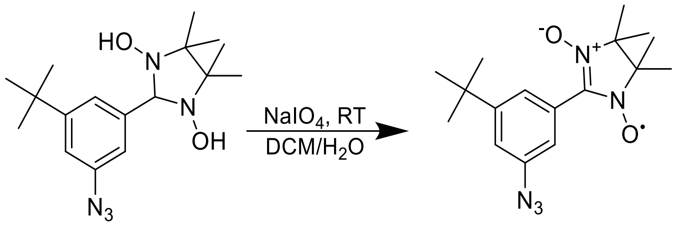

2c **2**

Compound **2c** (250 mg, 0.749 mmol) was dissolved in degassed dichloromethane (CH_2Cl_2, 50 mL) and a solution of $NaIO_4$ (200 mg, 0.973 mmol, 1.25 eq.) in degassed water (50 mL) was added dropwise over 25 min while vigorously stirring under N_2. The organic layer slowly turned deep blue and the mixture was left to stir for 1 h. The progression of the reaction was monitored via TLC (eluent: CH_2Cl_2). The reaction mixture was the diluted with 50 mL of CH_2Cl_2 and the layers separated. The organic phase was then washed with distilled water (2 × 100 mL), brine (100 mL), dried over $MgSO_4$, filtered and the solvent removed *in vacuo*. The product was purified via column chromatography (SiO_2, eluent: petroleum ether/EtOAc 9:1) to yield radical **2** as blue solid (197 mg, 80%). ESI-MS (+, *m/z*, %): 331.5 [$M+H^+$,100], 353.4 [$M+Na^+$,1], 371.4 [$M+K^+$,3]; UV/Vis, λ/nm (MeCN), (ε, M^{-1} cm^{-1}): 583 (222), 365 (8087). EPR: a_N= 7.5 G, MeCN. Anal. Calcd for $C_{17}H_{24}N_5O_2$: C, 61.80; H, 7.32; N, 21.20. Found: C, 62.22; H, 7.44; N, 20.95.

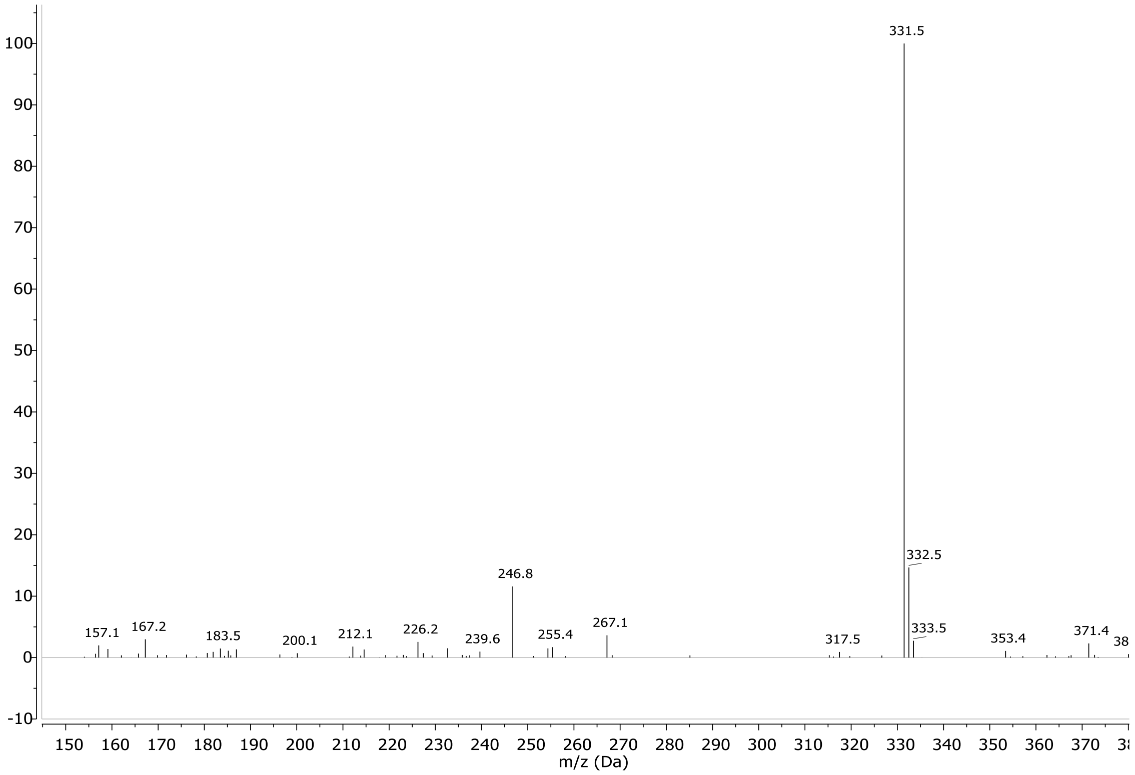

Figure S11. MS spectrum (ESI +) of compound **2**.

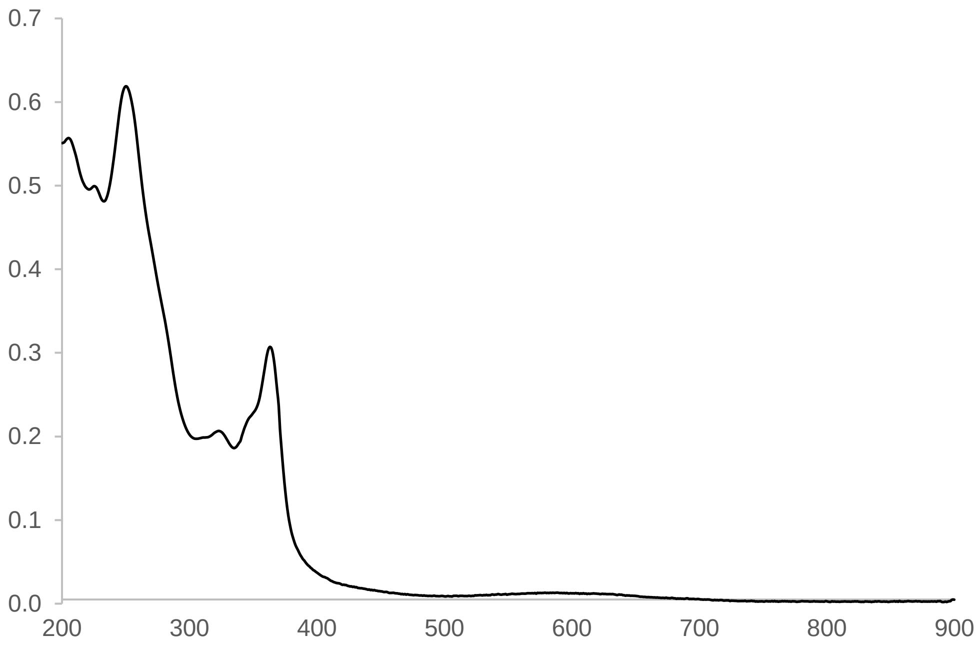

Figure S12. UV-Vis spectra of compound **2** (0.038 mM) in MeCN.

Synthesis of 2-(3-(*tert*-butyl)-5-(1,3-dioxolan-2-yl)phenyl)-6-chloropyridine (3a)

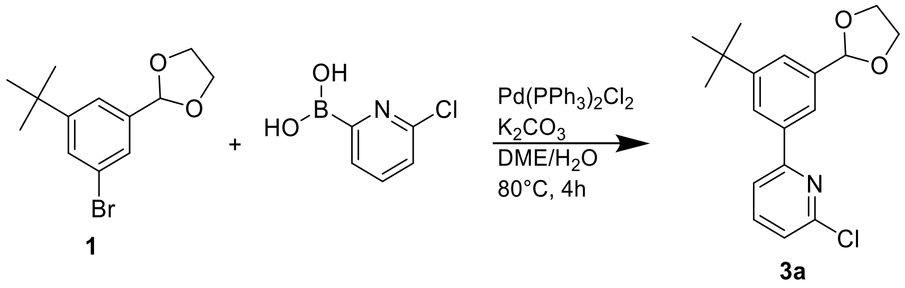

To compound **1** (244 mg, 0.86 mmol), (6-chloropyridin-2-yl)boronic acid (136 mg, 0.86 mmol, 1 eq.), $Pd(PPh_3)_2Cl_2$ (12 mg, 0.017 mmol, 2% mol.) degassed DME (3 mL) was added and the suspension degassed again by bubbling Ar through the mixture. A degassed solution of K_2CO_3 (474 mg, 3.42 mmol, 4 eq.) was added and the solution slowly heated to 80 °C. The mixture was stirred 4h at 80°C and the progress of the reaction checked via TLC (eluent: cyclohexane/CH_2Cl_2 9:1). The reaction mixture was then cooled to RT, diluted with water (15 mL) and extracted with Et_2O (3 × 15mL), washed with brine (45 mL), dried on $MgSO_4$, filtered and the solvent removed *in vacuo* to yield a brown oil. The crude mixture was purified via column chromatography (SiO_2, eluent: cyclohexane/EtOAc 9:1) to yield compound **3a** as a yellow solid (154 mg, 57%). ^1H NMR (400 MHz, CD_3CN) δ = 8.06 (m, 1H) 7.91 (m, 1H), 7.87-7.80 (m, 2H), 7.60 (m, 1H), 7.36-7.34 (m, 1H), 5.80 (s, 1H), 4.13-4.01 (m, 4H), 1.37 (s, 9H). ^{13}C NMR (101 MHz, CD_3CN) δ = 275.56, 270.01, 268.69, 258.11, 256.93, 255.49, 242.97, 242.42, 237.23, 221.37, 183.14, 152.58, 148.47, 118.26. GC-MS (E.I., *m/z,* %): 315.8 [M-$H_2^{+\bullet}$ 11], 280.9 [(M-Cl-2H),$^+$ 30].

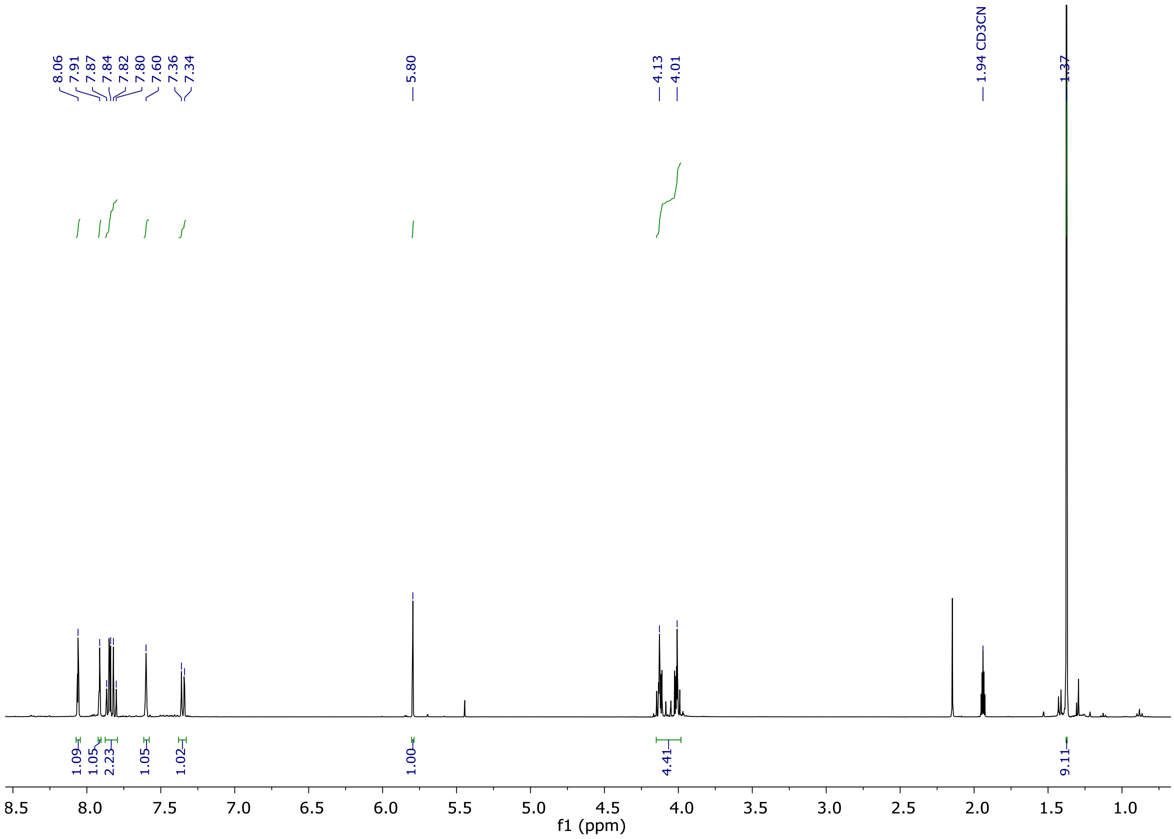

Figure S13 ^{1}H-NMR (400MHz) of **3a** in CD_3CN at 298K.

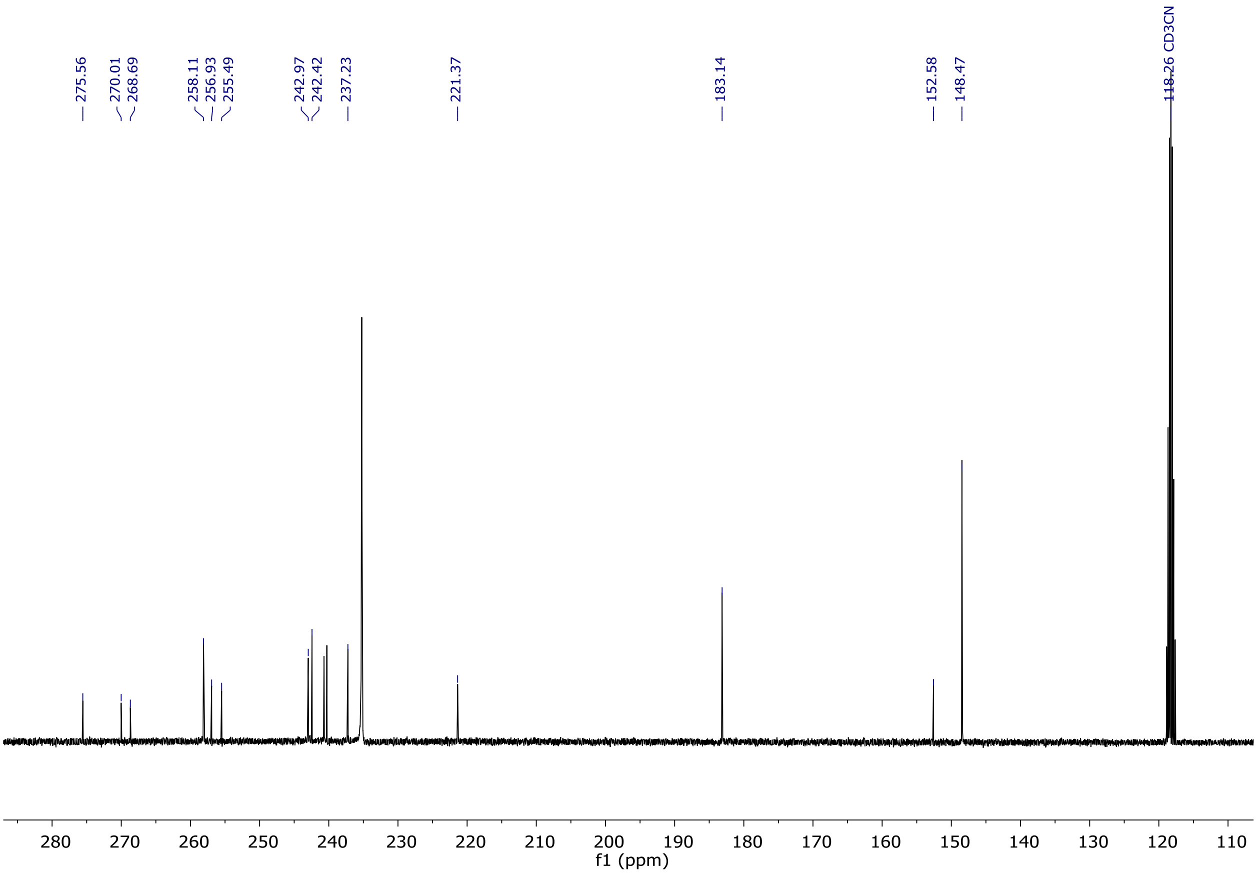

Figure S14 ^{13}C-NMR (101MHz) of **3a** in DMSO-d_6 at 298K.

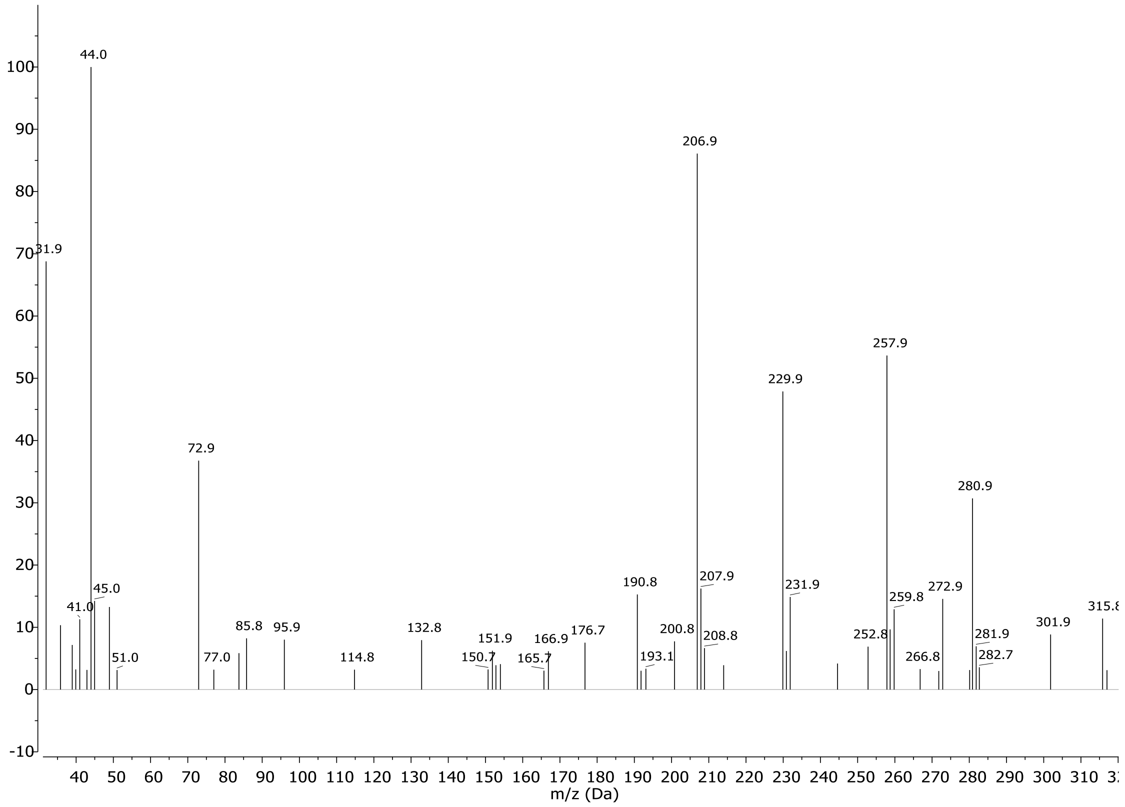

Figure S15. Mass spectrum (EI) of compound **3a.**

Synthesis of 3-(*tert*-butyl)-5-(6-((trimethylsilyl)ethynyl)pyridin-2-yl)benzaldehyde (3b)

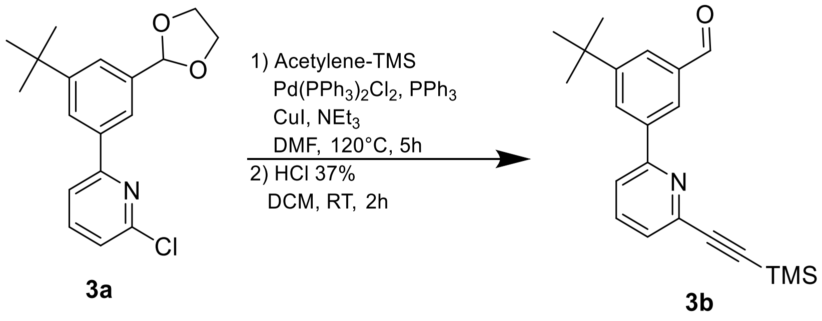

Compound **3a** (250 mg, 0.786 mmol), $Pd(PPh_3)_2Cl_2$ (13.3 mg, 0.019 mmol, 2.5% mol.), PPh_3 (2.57 mg, 9.82 µmol, 1.25%), CuI (1.49 mg, 7.86 µmol, 1%) were added to a 10 ml DMF that was then flushed with Ar. Degassed dry DMF (4 mL) was added and the mixture degassed again by bubbling Ar through it. NEt_3 (220 µL, 1.57 mmol, 2 eq.) was added followed by TMS-acetylene (544 µL, 3.93 mmol, 5 eq.). the mixture was stirred at RT for 1 h and then heated to 120 °C for 5 h. the reaction was monitored via TLC (eluent: cyclohexane/EtOAc 9:1). The reaction mixture was then cooled to RT and the DMF evaporated in vacuo to yield a brown oil that was filtered through silica (length ~ 5 cm, eluent: Et_2O). The crude mixture was purified via column chromatography (SiO_2, eluent: cyclohexane/EOtAc 9:1) to yield the protected aldehyde **3b** as a brown oil (231 mg, 79%). ^1H NMR (400 MHz, CD_3CN) δ = 8.05 (m, 1H)

7.92(m, 1H), 7.88-7.79 (m, 2H), 7.59 (m, 1H), 7.45-7.43 (m, 1H), 5.80 (s, 1H), 4.15-3.99 (m, 4H), 1.38 (s, 9H), 0.29 (s, 9H). The protected aldehyde (231 mg, 0.608 mmol) was dissolved in CH_2Cl_2 (5 mL) and HCl 37% (1 eq.) was added. The mixture was stirred at RT for 2 h. To the mixture was added $NaHCO_3$ (50 mL) and extracted with CH_2Cl_2 (3 × 50 mL). The organic phase was washed twice with water and with brine, dried on $MgSO_4$, filtered and the solvent removed *in vacuo*. the crude mixture was purified via column chromatography (SiO_2, eluent: cyclohexane/EtOAc 9:1) to yield compound **3b** as a yellowish solid (195 mg, 96%). ^{1}H NMR (400 MHz, CD_3CN) δ = 10.07 (s, 1H), 8.30 (m, 2H), 8.00 (m, 1H), 7.91-7.81 (m, 2H), 7.47 (m, 1H), 1.40 (s, 9H), 0,28 (s, 9H). ^{13}C NMR (101 MHz, CD_3CN) δ = 193.71, 157.28, 153.92, 143.55, 140.26, 138.63, 138.10, 130.51, 127.98, 127.50, 126.43, 121.58, 105.03, 95.13, 35.76, 31.42, 1.32, -0.28. GC-MS (E.I., *m/z*, %): 335 [$M^{+\bullet}$, 45], 320 [M-CH_3,$^+$ 100].

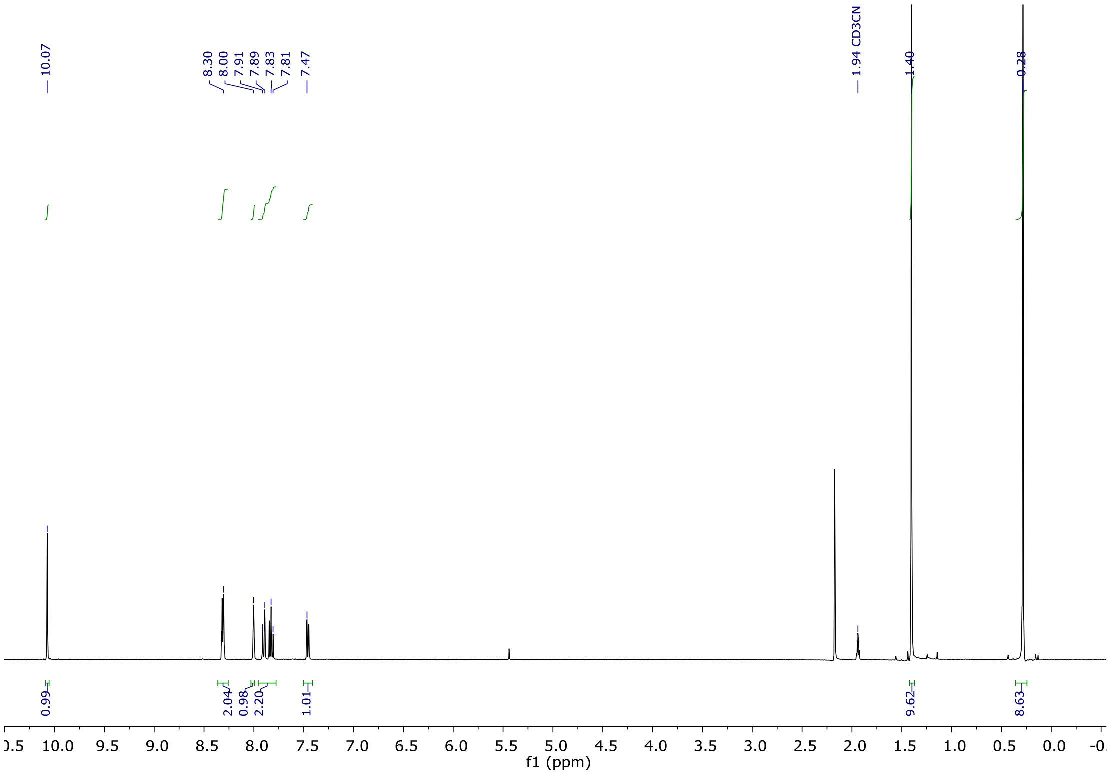

*__Figure S16.__ ^{1}H-NMR (400MHz) of **3b** in CD_3CN at 298K.

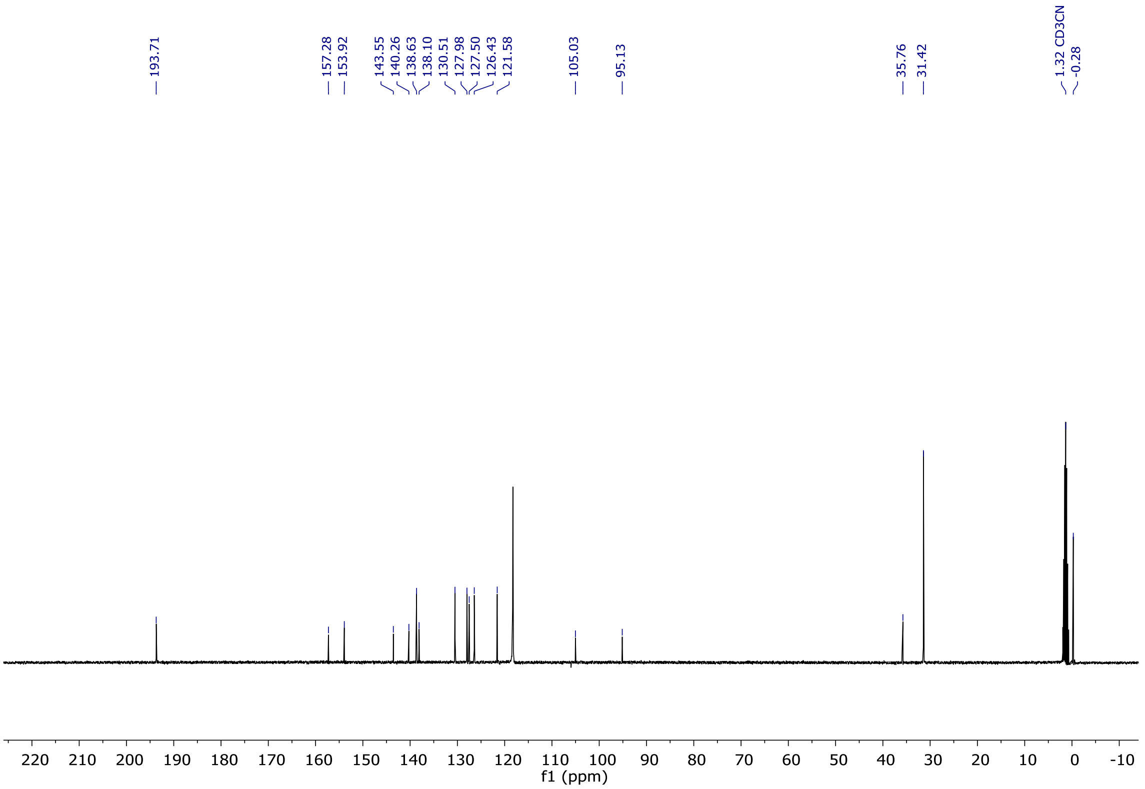

Figure S17. ^{13}C-NMR (101 MHz) of **3b** in CD_3CN at 298K.

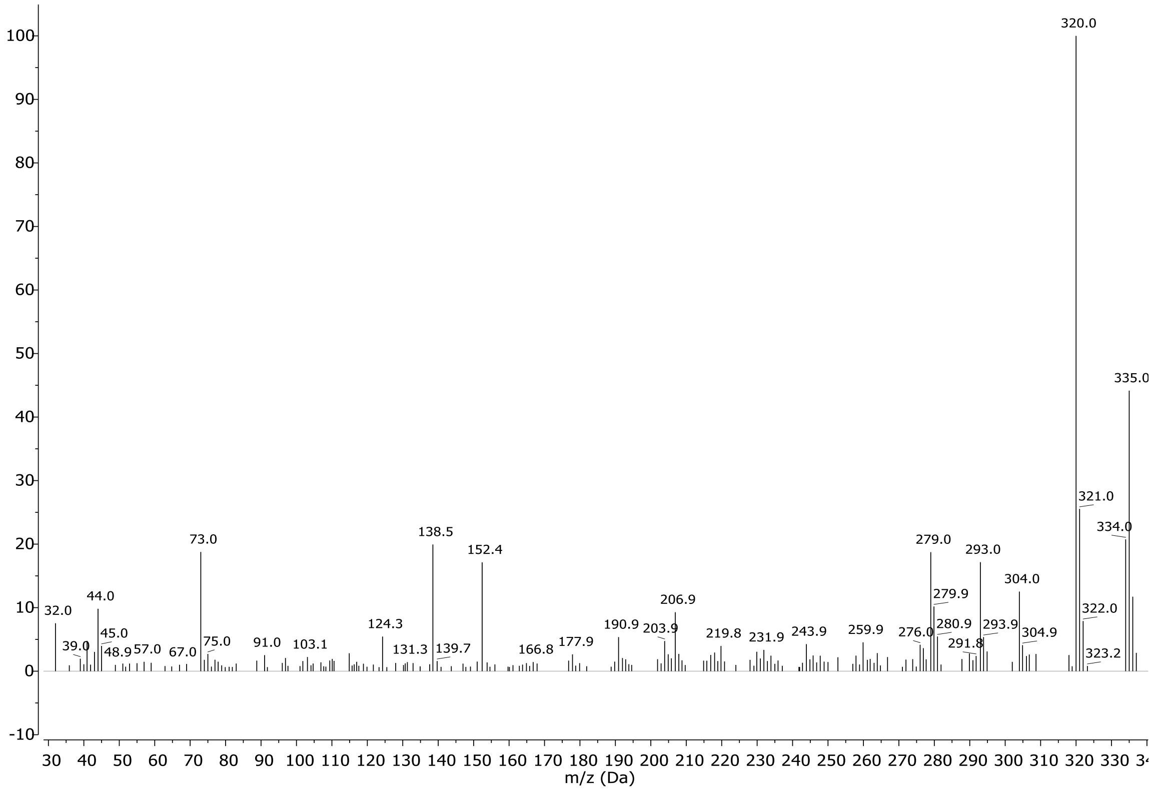

Figure S18. Mass spectrum (EI) of compound **3b**.

Synthesis of 2-(3-(*tert*-butyl)-5-(6-((trimethylsilyl)ethynyl)pyridin-2-yl)phenyl)-4,4,5,5-tetramethylimidazolidine-1,3-diol (3c)

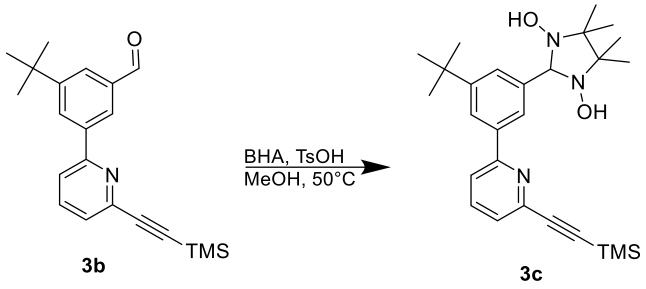

A degassed solution of aldehyde **3b** (35 mg, 0.104 mmol), BHA (15.5 mg, 0.104 mmol, 1 eq.), TsOH (1 mg, 5.25 µmol, 5% mol.) in dry MeOH (1 mL) was stirred under N_2 at 50 °C for 18 h. The cloudy reaction mixture was then cooled to RT, concentrated in vacuo to a minimal volume and the product precipitated with pentane, filtered and washed again with pentane to yield **3c** as a white powder (32 mg, 65%) that was used without further purification in the next step. ^{1}H NMR (400 MHz, DMSO-d_6) δ = 7.96-7.87 (m, 4H), 7.81 (s, 2H, HO-N), 7.63 (m, 1H), 7.51-7.49 (m, 1 H), 4.61 (s, 1 H), 1.36 (s, 9H), 1.12-1.10 (ds, 12H), 0.29 (s, 9H). ^{13}C NMR (101 MHz, $CDCl_3$) δ = 157.41, 150.44, 142.23, 141.72, 137.74, 137.13, 126.50, 125.97, 124.46, 122.58, 120.57, 104.73, 93.74, 90.70, 66.18, 39.52, 34.57, 31.29, 24.41, 17.15, -0.30. LC-MS (ESI +, *m/z*, %): 466.6 [M+H$^+$, 68], 488.6 [M+Na$^+$, 100], 504.6 [M+K$^+$, 10].

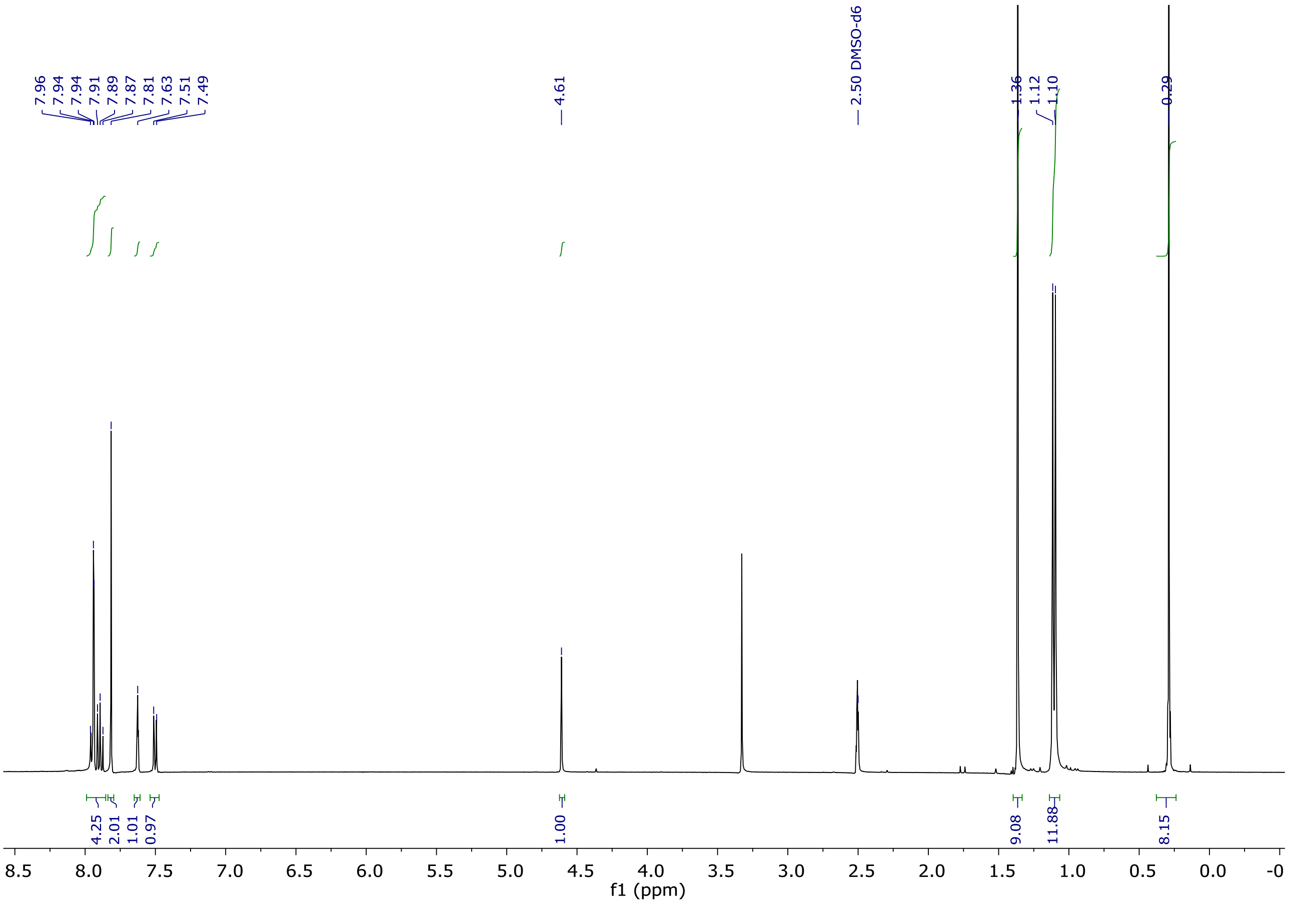

Figure S19. ^{1}H-NMR (400 MHz) of **3c** in DMSO-d_6 at 298K.

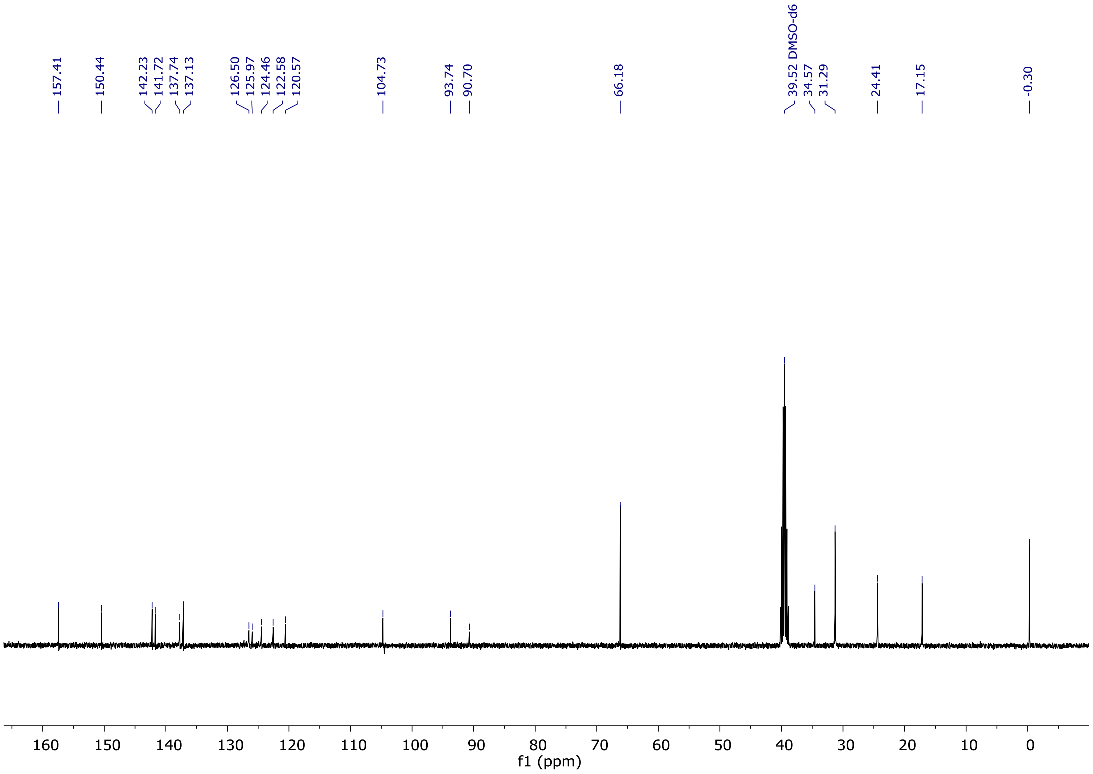

Figure S20. ^{13}C-NMR (101 MHz) of **3c** in CD_3CN at 298K.

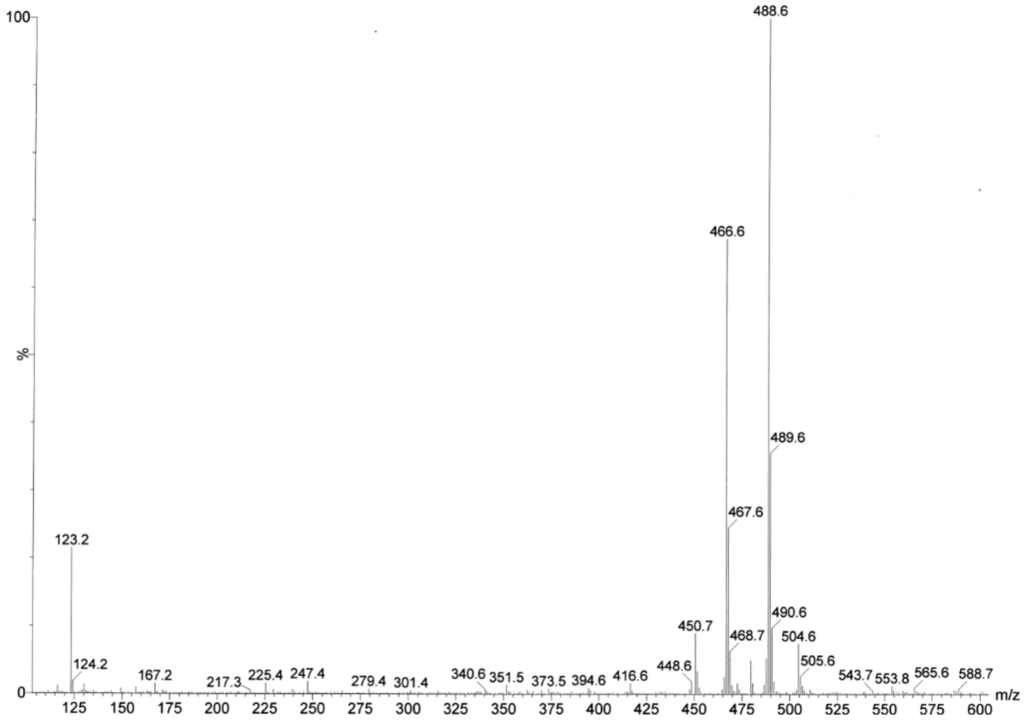

Figure S21. Mass spectrum (ESI +) of **3c** in MeOH.

Synthesis of 1-(4,4,5,5-tetramethyl-1-oxyl-3-oxide- imidazoline-2-yl) 3-(*tert*-butyl)-5-(6-((trimethylsilyl)ethynyl)pyridin-2-yl)-benzene (3d)

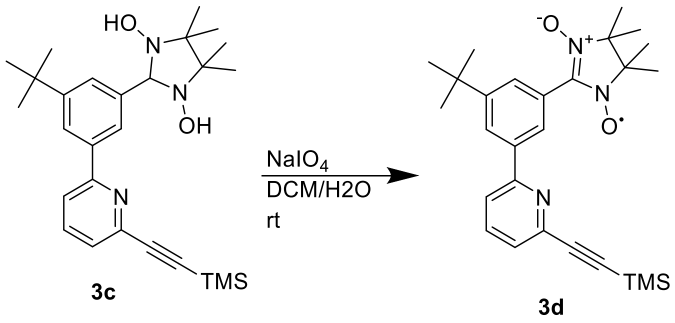

Compound **3c** (32 mg, 0.0687 mmol) was dissolved in degassed CH_2Cl_2 (5 mL) and then a solution of $NaIO_4$ (18.53 mg, 0.0859 mmol, 1.25 eq.) in degassed water (5 mL) was added dropwise over 25 min while vigorously stirring under N_2. The organic layer slowly turned deep blue and the mixture was left to stir for 1 h. The progression of the reaction was monitored via TLC to monitor the disappearance of the starting material (eluent: CH_2Cl_2/EtOAc 98:2). The reaction mixture was the diluted with 15 mL of CH_2Cl_2 and 15 mL of H_2O and the layers separated. The organic phase was then washed with distilled water (2 × 20 mL), brine (20 mL), dried over $MgSO_4$, filtered and the solvent removed *in vacuo*. The product was purified via column chromatography (SiO_2, eluent: CH_2Cl_2 then CH_2Cl_2/EtOAc 98:2) to yield radical **3d** as blue solid (21 mg, 66%). ESI-MS (+, *m/z*, %): 464 [M+2H$^+$, 100], 486 [M+Na$^+$, 90]. EPR: a_N= 7.55 G, MeCN.

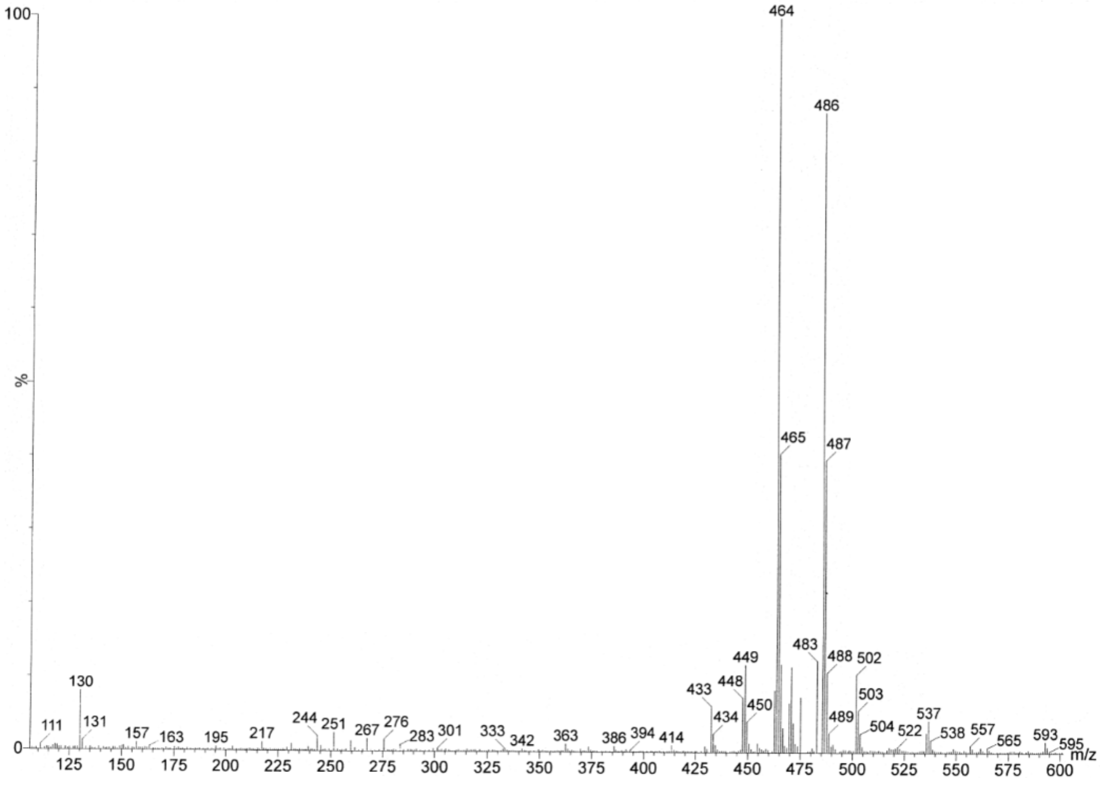

Figure S22. Mass spectrum (ESI +) of compound **3d**.

Synthesis of 1-(4,4,5,5-tetramethyl-1-oxyl-3-oxide-imidazoline-2-yl) 3-(*tert*-butyl)-5-(6-(ethynyl)pyridin-2-yl)-benzene (3)

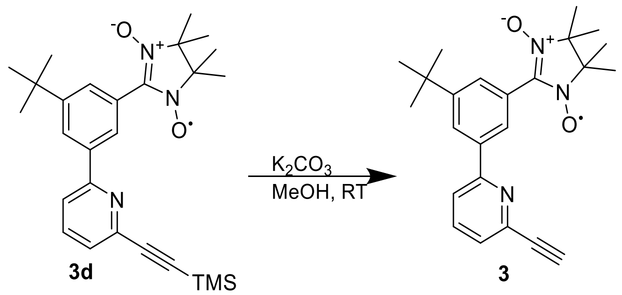

Radical **3d** (21 mg, 0.0454 mmol) was dissolved in degassed MeOH (1 mL) and K_2CO_3 (18 mg, 0.13 mmol, 3 eq.) was added. The reaction mixture was stirred overnight under nitrogen. After this time the suspension was filtered, MeOH removed *in vacuo* and re suspended in CH_2Cl_2 (50 mL). The organic layer was washed with sat. $NaHCO_3$ (50mL), water (50 mL) and then brine (50 mL). the solution was dried on $MgSO_4$, filtered and the solvent removed *in vacuo*. The crude was purified on silica gel chromatography (eluent: CH_2Cl_2 then CH_2Cl_2/EtOAc 9:1) to yield radical **3** as a blue solid (17 mg, 98%). ESI-MS (+, *m/z*, %): 392 [$MH+H^+$, 50], 414 [$MH+Na^+$,100], 430 [$MH+K^+$, 12]. EPR: a_N= 7.53 G, MeCN. Anal. Calcd for $C_{24}H_{28}N_3O_2$: C, 73.82; H, 7.23; N, 10.76. Found: C, 73.52; H, 7.20; N, 10.95.

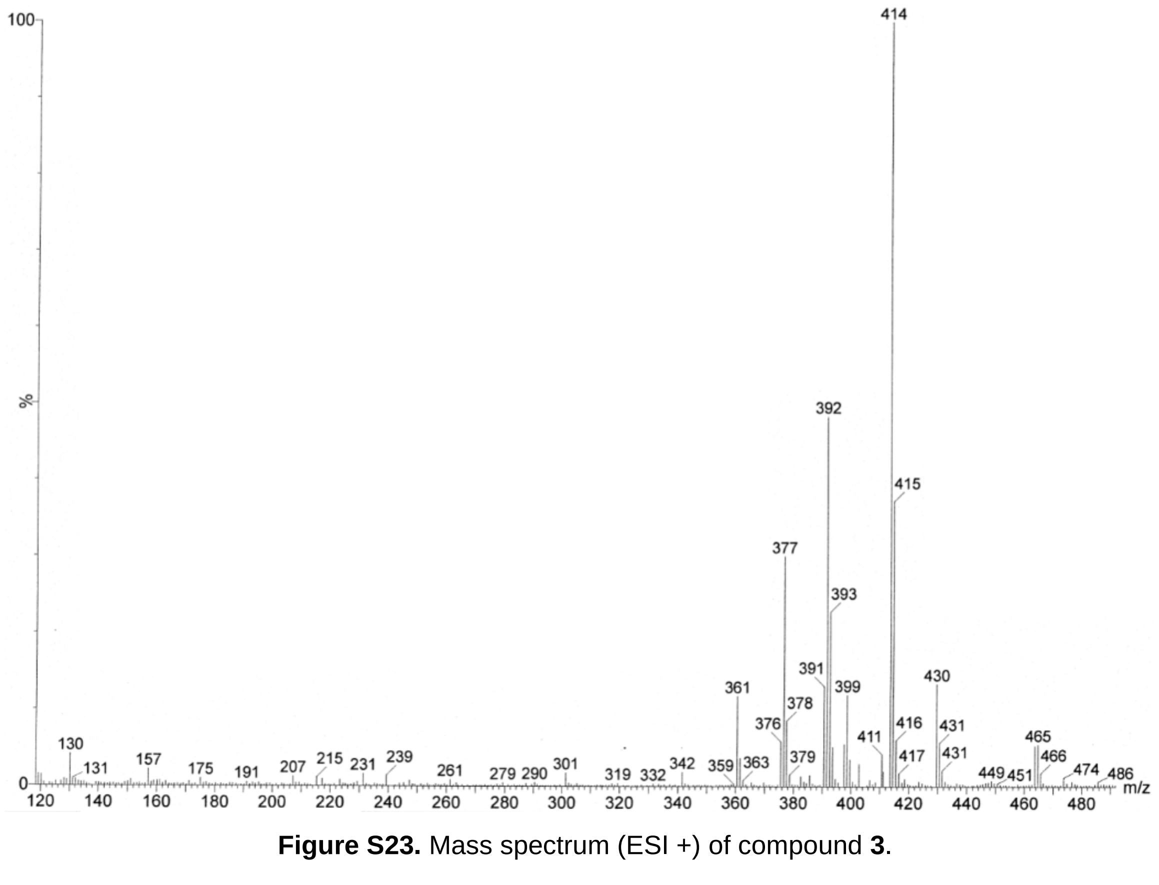

Figure S23. Mass spectrum (ESI +) of compound **3**.

Synthesis of Rotax-1

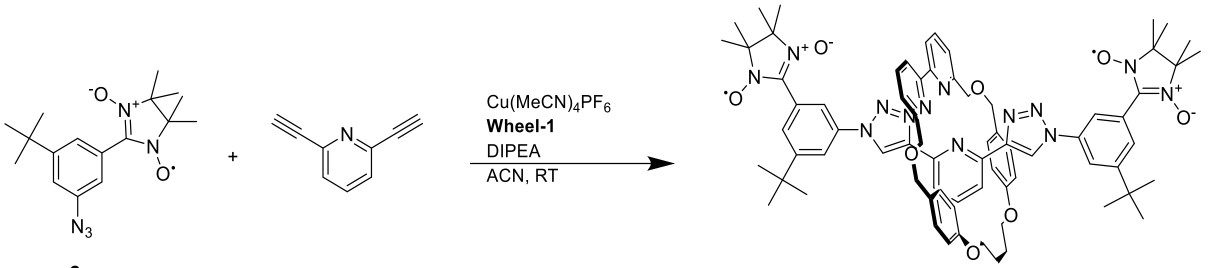

2 **Rotax-1**

A solution of radical **2** (20 mg, 0.0605 mmol) and 2,6-diethynylpyridine (3.83 mg, 0.0302 mmol, 1eq.) in degassed dry MeCN (1mL) was transferred to a solution of $Cu(MeCN)_4PF_6$ (10.8 mg, 0.0290 mmol, 0.96 eq.) and macrocycle **Wheel-1** (14.57 mg, 0.0302 mmol, 1 eq.) in degassed dry MeCN (1 mL). DIPEA (11 µL, 0.0605 mmol, 2 eq.) was added and the mixture stirred under N_2 for 18h. the progress of the reaction was monitored via TLC (eluent: CH_2Cl_2/EtOAc 8:2). When the reaction was complete the mixture was diluted with 100 mL of CH_2Cl_2 and 100 mL of $EDTA_{sat}/NH_3$ 17,5% solution was added. The bilayer mixture was stirred for an additional hour and then the layers were separated. The organic layer was washed with water (2 × 100 mL), brine (100 mL) and dried over $MgSO_4$. Filtration and evaporation of the solvent led to a greenish solid that was purified via column chromatography (SiO_2, eluent: CH_2Cl_2/EtOAc 9:1, 8:2 and 7:3) to yield **Rotax-1** as a blue solid (32 mg, 82%). ESI-MS (+, *m/z*, %): 636.5 [MH+2H$^+$, 100],1272.1 [MH+H$^+$, 60], 1293.1 [M+Na$^+$, 8]. UV/Vis (MeCN), λ/nm (ε, M^{-1} cm^{-1}): 581 (815), 366 (22137). EPR: a_N= 7.33 G, toluene. Anal. Calcd for $C_{73}H_{83}N_{13}O_8$: C, 69.01; H, 6.58; N, 14.33. Found: C, 69.19; H, 6.55; N, 14.28.

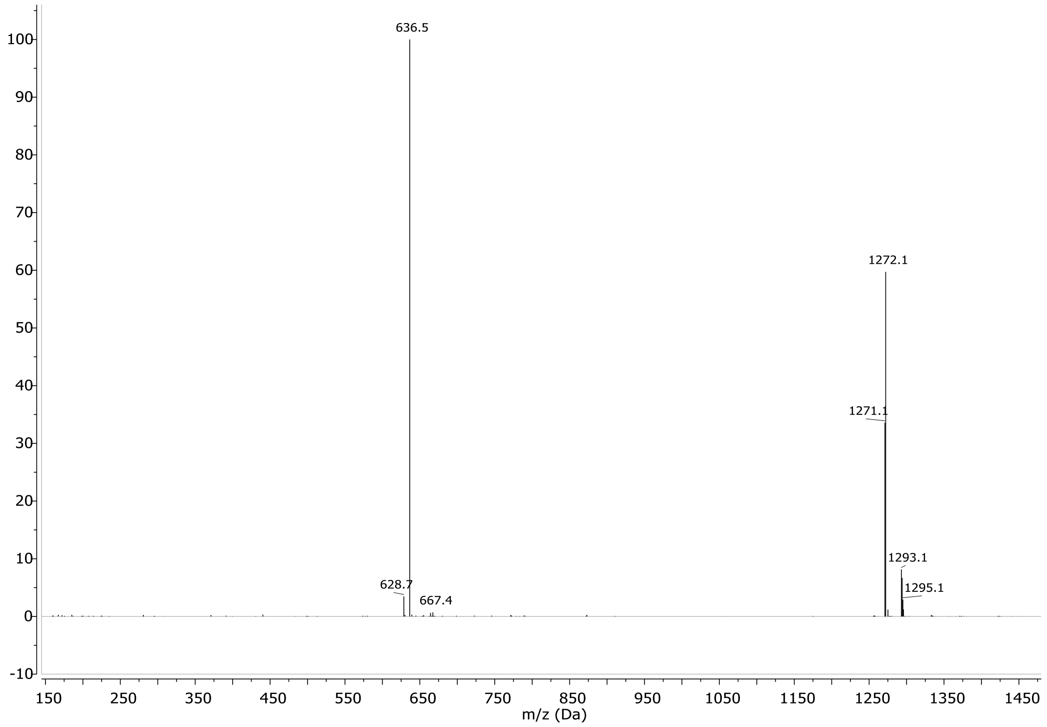

Figure S24. Mass spectrum (ESI +) of Rotaxane **Rotax-1.**

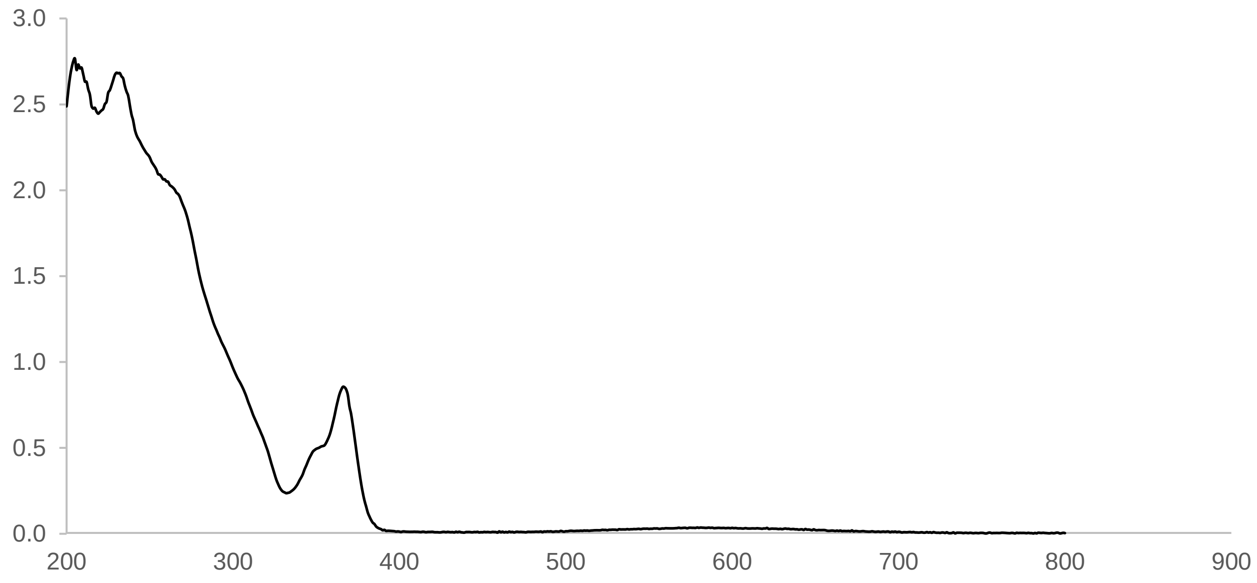

Figure S25. UV-Vis spectra of rotaxane **Rotax-1** (0.036 mM) in MeCN.

Synthesis of Rotax-2

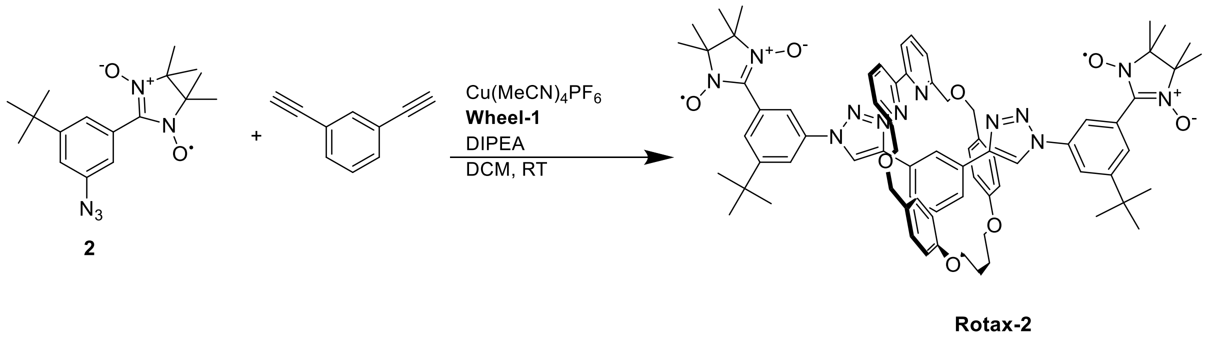

A solution of radical **2** (20 mg, 0,0605 mmol. 2 eq.) and 2,6-diethynylbenzene (3.81 mg, 0.0302 mmol, 1eq.) in degassed dry CH_2Cl_2 (1 mL) was transferred to a solution of $Cu(MeCN)_4PF_6$ (10.8 mg, 0.0290 mmol, 0.96 eq.) and macrocycle **Wheel-1** (14.57 mg, 0.0302 mmol, 1 eq.) in degassed dry CH_2Cl_2 (1 mL). DIPEA (11 µL, 0.0605 mmol, 2 eq.) was added and the mixture stirred under N_2 for 72h. the progress of the reaction was monitored via TLC (eluent: CH_2Cl_2/EtOAc 8:2). When the reaction was complete the mixture was diluted with 100 mL of CH_2Cl_2 and 100 mL of $EDTA_{sat}/NH_3$ 17,5% solution was added. The bilayer mixture was stirred for an additional hour and then the layers were separated. The organic layer was washed with water (2 × 100 mL), brine (100 mL) and dried over $MgSO_4$. Filtration and evaporation of the solvent led to a greenish solid that was purified via column chromatography (SiO_2, eluent: CH_2Cl_2/EtOAc 9:1 and 7:3) to yield **Rotax-2** as a blue solid (10 mg, 26%). ESI-MS (+, *m/z*, %): 1292 [M+Na$^+$, 100], UV/Vis (MeCN), λ/nm (ε, M^{-1} cm^{-1}): 579 (780), 366 (18146). EPR: a_N= 7.35 G, toluene. Anal. Calcd for $C_{74}H_{84}N_{11}O_8$: C, 70.01; H, 6.67; N, 13.24. Found: C, 70.18; H, 6.65; N, 13.12.

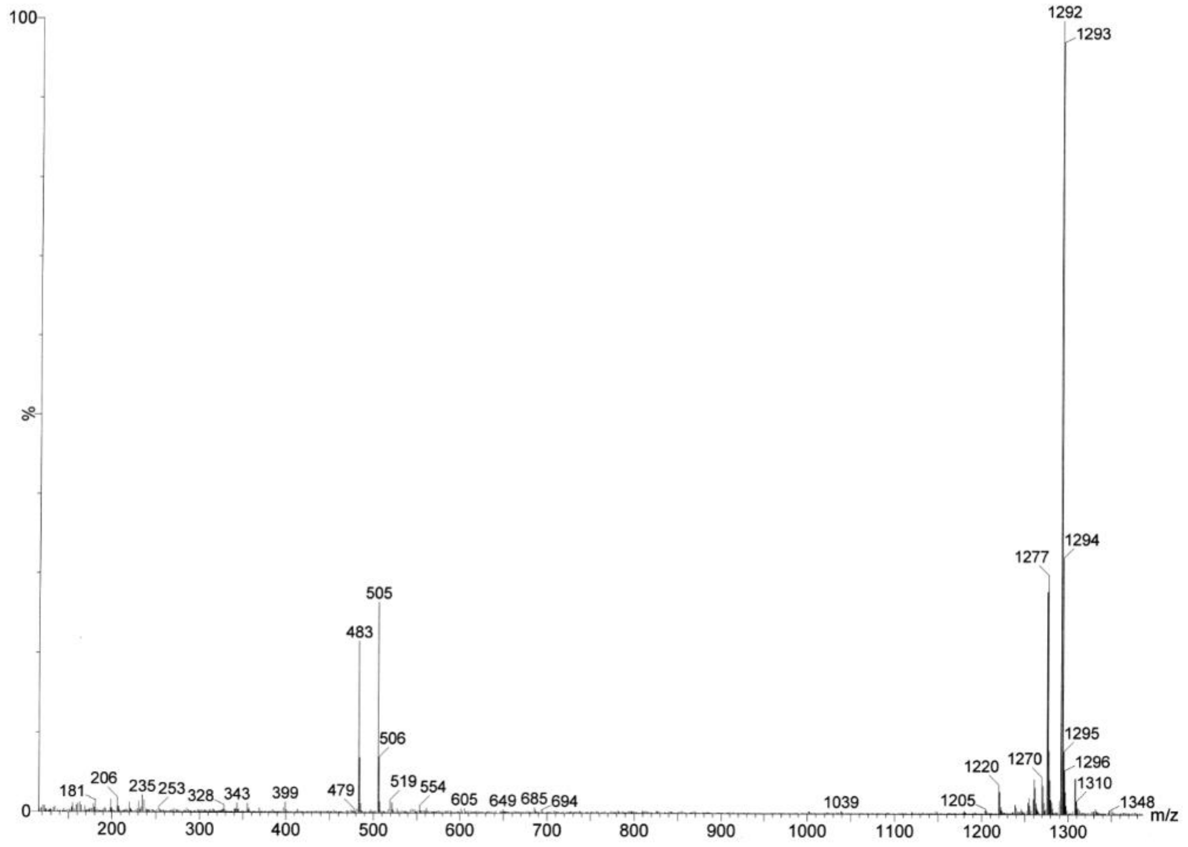

Figure S26. Mass spectrum (ESI +) of rotaxane **Rotax-2**.

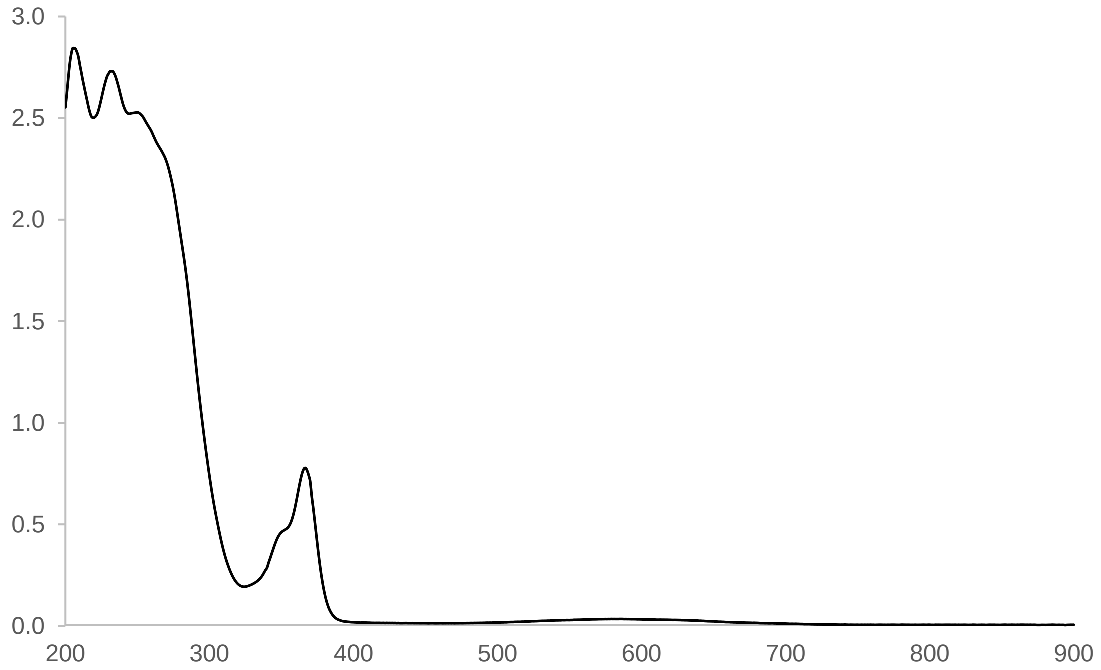

Figure S27. UV-Vis spectra of rotaxane **Rotax-2** (0.040 mM) in MeCN.

Synthesis of Rotax-3

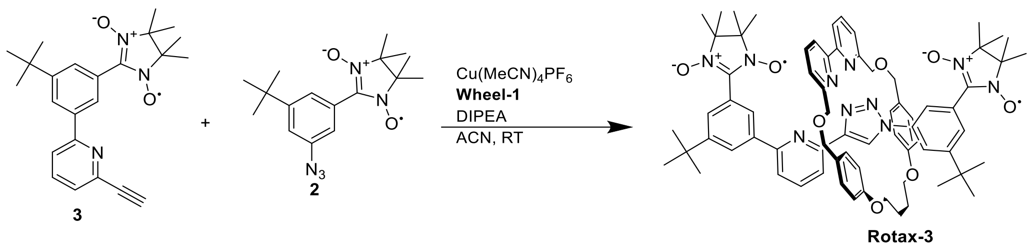

A solution of radical **3** (7 mg, 0.0179 mmol) and **2** (5.92 mg, 0.0179 mmol, 1eq.) in degassed dry MeCN (0.5 mL) was transferred to a solution of $Cu(MeCN)_4PF_6$ (6.40 mg, 0.0172 mmol, 0.96 eq) and macrocycle **Wheel-1** (8.63 mg, 0.0179 mmol, 1 eq) in degassed dry MeCN (0.5 mL). DIPEA (7 µL, 0.0358 mmol, 2 eq) was added and the mixture stirred under N_2 for 18h. the progress of the reaction was monitored via TLC (eluent: CH_2Cl_2/EtOAc 9:1). When the reaction was complete the mixture was diluted with 25 mL of CH_2Cl_2 and 25 mL of $EDTA_{sat}/NH_3$ 17,5% solution was added. The bilayer mixture was stirred for an additional hour and then the layers were separated. The organic layer was washed with water (2 × 25 mL), brine (25 mL) and dried over $MgSO_4$. Filtration and evaporation of the solvent led to a greenish solid that was purified via column chromatography (SiO_2, eluent: CH_2Cl_2/EtOAc 9:1 then 8:2) to yield **Rotax-3** as a blue solid (18 mg, 85%). ESI-MS (+, *m/z*, %): 1204 [$M+H^+$, 65], 1226 [$M+Na^+$, 100]. UV/Vis, λ/nm (MeCN), (ε, M^{-1} cm^{-1}): 589 (813), 365 (17383). EPR: a_N= 7.41 G, toluene. Anal. Calcd for $C_{71}H_{82}N_{10}O_8$: C, 70.86; H, 6.87; N, 11.64. Found: C, 70.42; H, 6.90; N, 11.72.

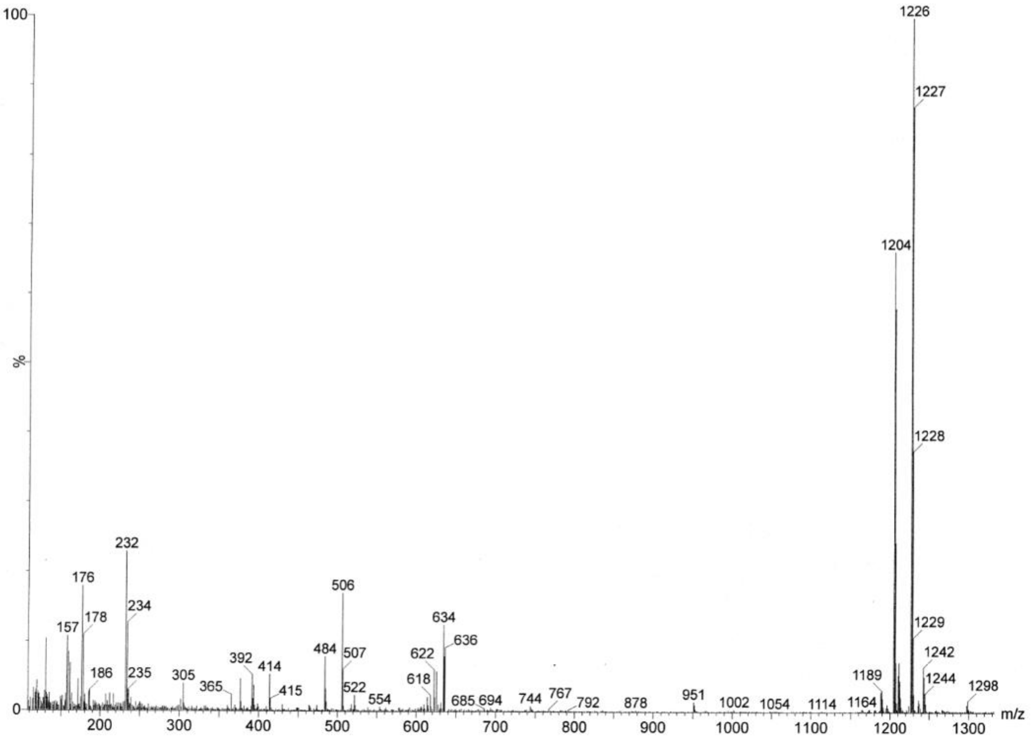

Figure S28. Mass spectrum (ESI +) of rotaxane **Rotax-3** recorded in MeOH.

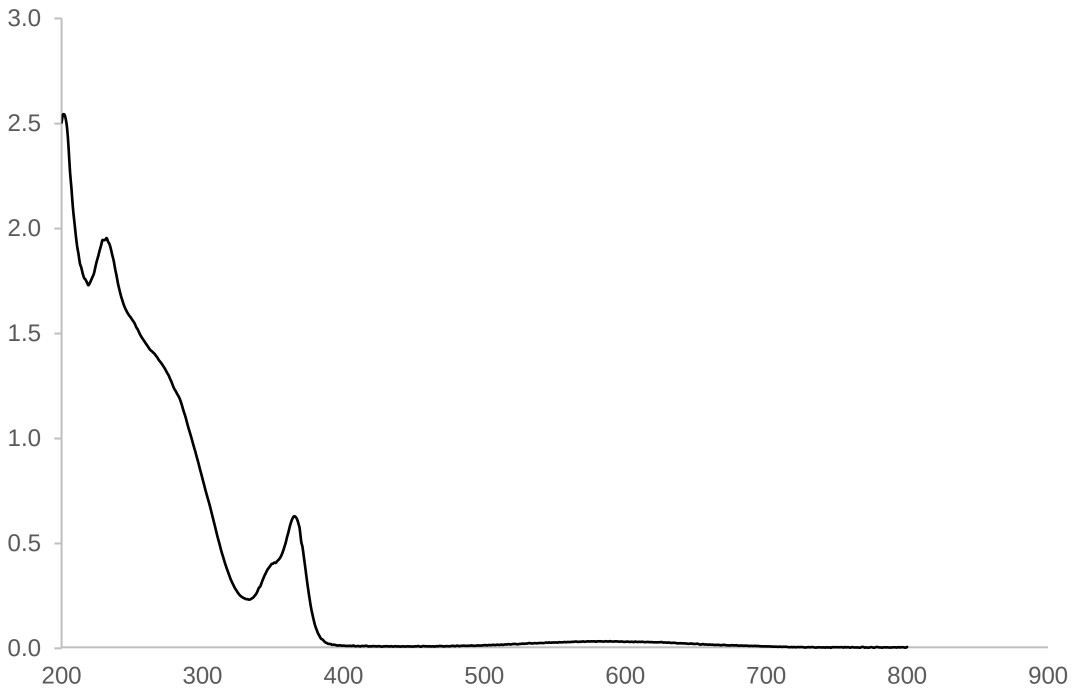

Figure S29. UV-Vis spectra of rotaxane **Rotax-3** (0.035 mM) in MeCN.

Synthesis of Axle-1

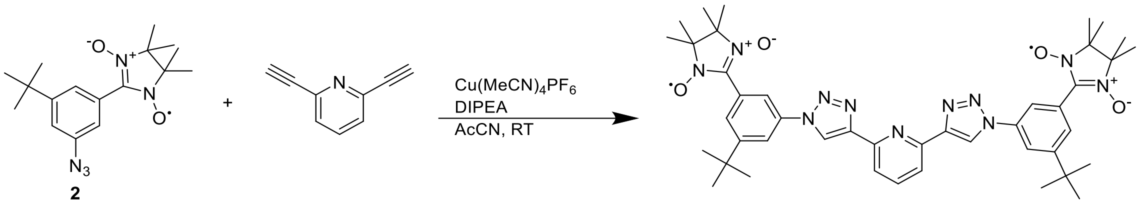

To a solution of radical **2** (20 mg, 0.0605 mmol) and 2,6-diethynylpyridine (3.84 mg, 0.0302 mmol, 1eq.) in degassed dry MeCN (2mL) was added $Cu(MeCN)_4PF_6$ (11.27.8 mg, 0.03026 mmol, 1 eq) and DIPEA (11 µL, 0.0605 mmol, 2 eq). The mixture stirred under N_2 for 18h. The mixture was then diluted with 25 mL of CH_2Cl_2 and 25 mL of $EDTA_{sat}/NH_3$ 17.5% solution was added. The bilayer mixture was stirred for an additional hour and then the layers were separated. The organic layer was washed with water (2 × 25 mL), brine (25 mL) and dried over $MgSO_4$. Filtration and evaporation of the solvent led to a greenish solid that was purified via column chromatography (SiO_2, eluent: CH_2Cl_2/EtOAc 7:3) to yield **Axle-1** as a blue solid (16 mg, 67%). ESI-MS (+, *m/z*, %): 788.7 [M+H$^+$, 100], 810.8 [M+Na$^+$, 25]. UV/Vis, λ/nm (MeCN), (ε, M^{-1} cm^{-1}): 588 (579), 366 (17583). EPR: a_N= 7.32 G, toluene. Anal. Calcd for $C_{43}H_{53}N_{11}O_4$: C, 65.54; H, 6.78; N, 19.55. Found: C, 65.88; H, 6.80; N, 19.25.

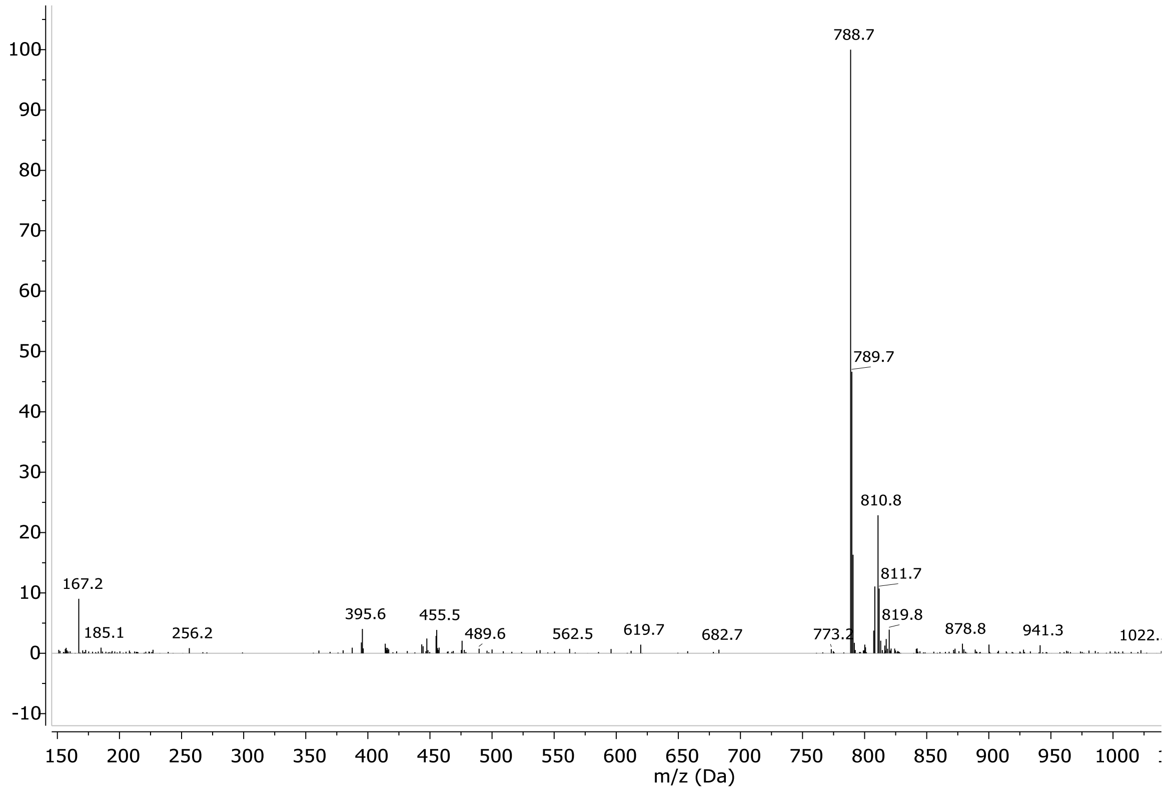

Figure S30. Mass spectrum (ESI +) of **Axle-1** recorded in MeOH.

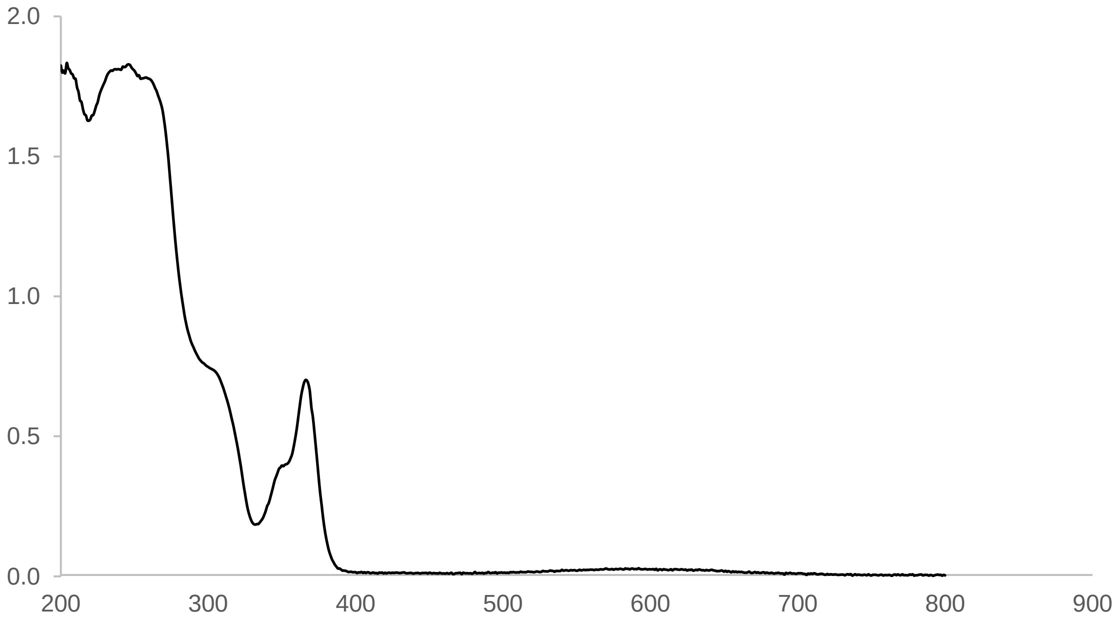

Figure S31. UV-Vis spectra of **Axle-1** (0.039 mM) in MeCN.

Synthesis of Axle-2

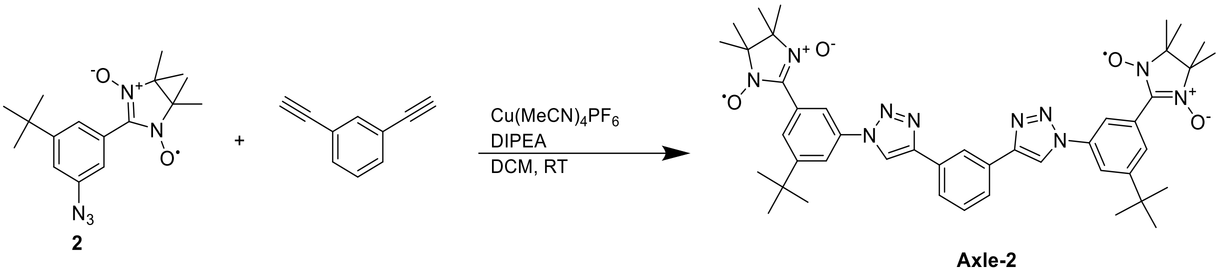

To a solution of radical **2** (20 mg, 0.0605 mmol) and 2,6-diethynylbenzene (3.81 mg, 0.0302 mmol, 1eq) in degassed dry CH_2Cl_2 (2mL) was added $Cu(MeCN)_4PF_6$ (11.27 mg, 0.0302 mmol, 1 eq) and DIPEA (11 µL, 0.0605 mmol, 2 eq.). The mixture stirred under N_2 for 18 h. The mixture was then diluted with 25 mL of CH_2Cl_2 and 25 mL of an $EDTA_{sat}/NH_3$ 17.5% solution was added. The bilayer mixture was stirred for an additional hour and then the layers were separated. The organic layer was washed with water (2 × 25 mL), brine (25 mL) and dried over $MgSO_4$. Filtration and evaporation of the solvent led to a greenish solid that was purified via column chromatography (SiO_2, eluent: CH_2Cl_2/EtOAc 8:2) to yield **Axle-2** as a blue solid (10 mg, 42%). ESI-MS (+, *m/z*, %): 810 [$M+Na^+$, 100]. UV/Vis, λ/nm (MeCN), (ε, M^{-1} cm^{-1}): 586 (736), 366 (20739). EPR: a_N= 7.39 G, toluene. Anal. Calcd for $C_{44}H_{54}N_{10}O_4$: C, 67.15; H, 6.92; N, 17.80. Found: C, 67.30; H, 6.89; N, 17.70.

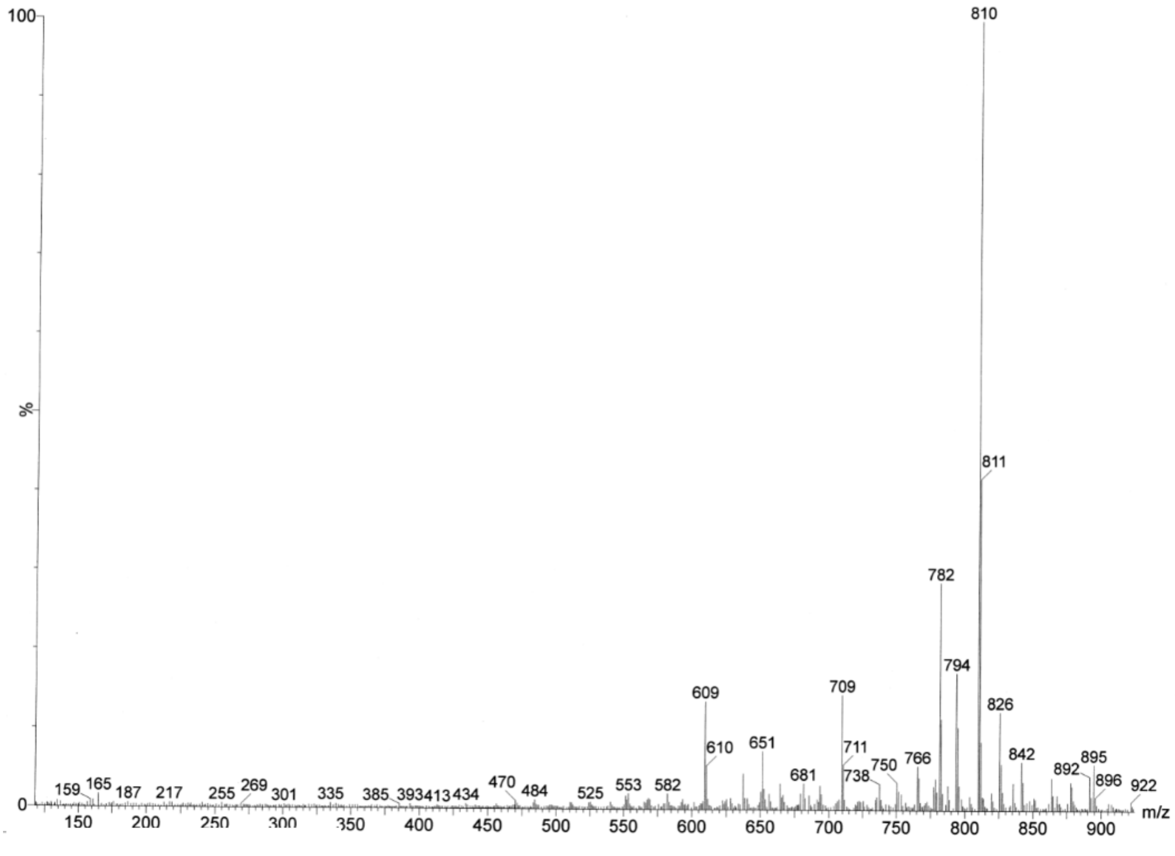

Figure S32. Mass spectrum (ESI +) of **Axle-2** recorded in MeOH.

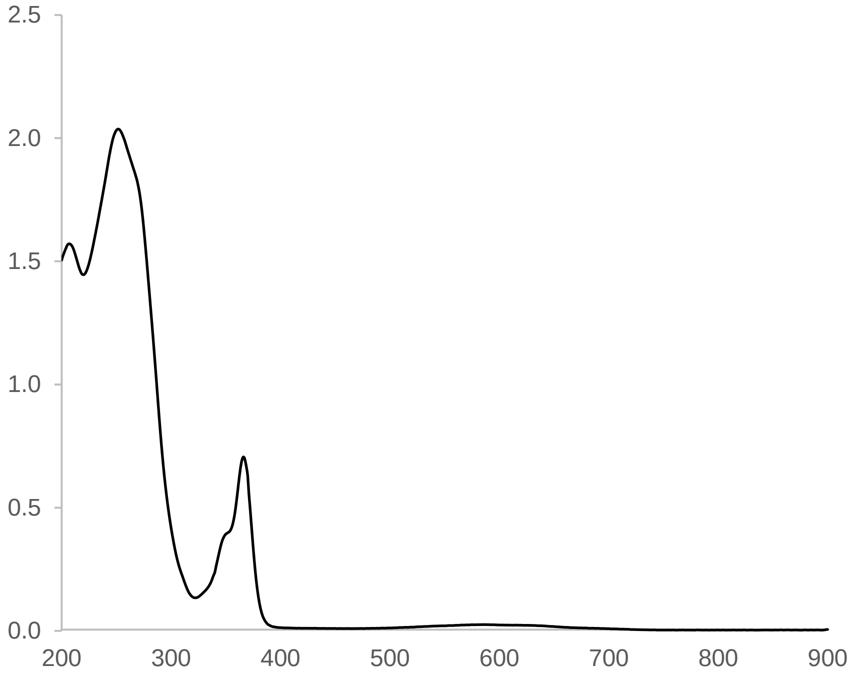

Figure S33. UV-Vis spectra of **Axle-2** (0.033 mM) in MeCN.

Synthesis of Axle-3

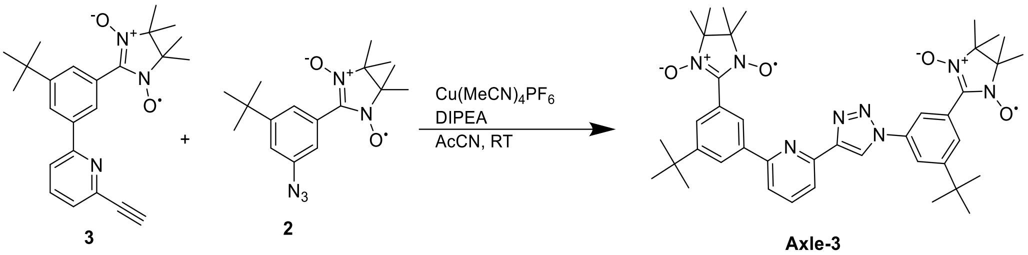

To a solution of radical **3** (7 mg, 0.0179 mmol) and **2** (5.92 mg, 0.0179 mmol, 1eq.) in degassed dry MeCN (1 mL) was added $Cu(MeCN)_4PF_6$ (6.67 mg, 0.0179 mmol, 1 eq.) and DIPEA (7 µL, 0.0358 mmol, 2 eq.). The mixture stirred under N_2 for 18h. The mixture was then diluted with 25mL of CH_2Cl_2 and 25 mL of $EDTA_{sat}/NH_3$ 17.5% solution was added. The bilayer mixture was stirred for an additional hour and then the layers were separated. The organic layer was washed with water (2 × 25 mL), brine (25 mL) and dried over $MgSO_4$. Filtration and evaporation of the solvent led to a greenish solid that was purified via column chromatography (SiO_2, eluent: CH_2Cl_2/EtOAc 8:2) to yield **Axle-3** as a blue solid (10.4 mg, 80%). ESI-MS (+, *m/z*, %): 722 [$M+H^+$], 744 [$M+Na^+$, 100]. UV/Vis, λ/nm (MeCN), (ε, M^{-1} cm^{-1}): 590 (845), 366 (18270). EPR: a_N= 7.32 G, toluene. Anal. Calcd for $C_{41}H_{52}N_8O_4$: C, 68.31; H, 7.27; N, 15.54. Found: C, 68.12; H, 7.30; N, 15.75.

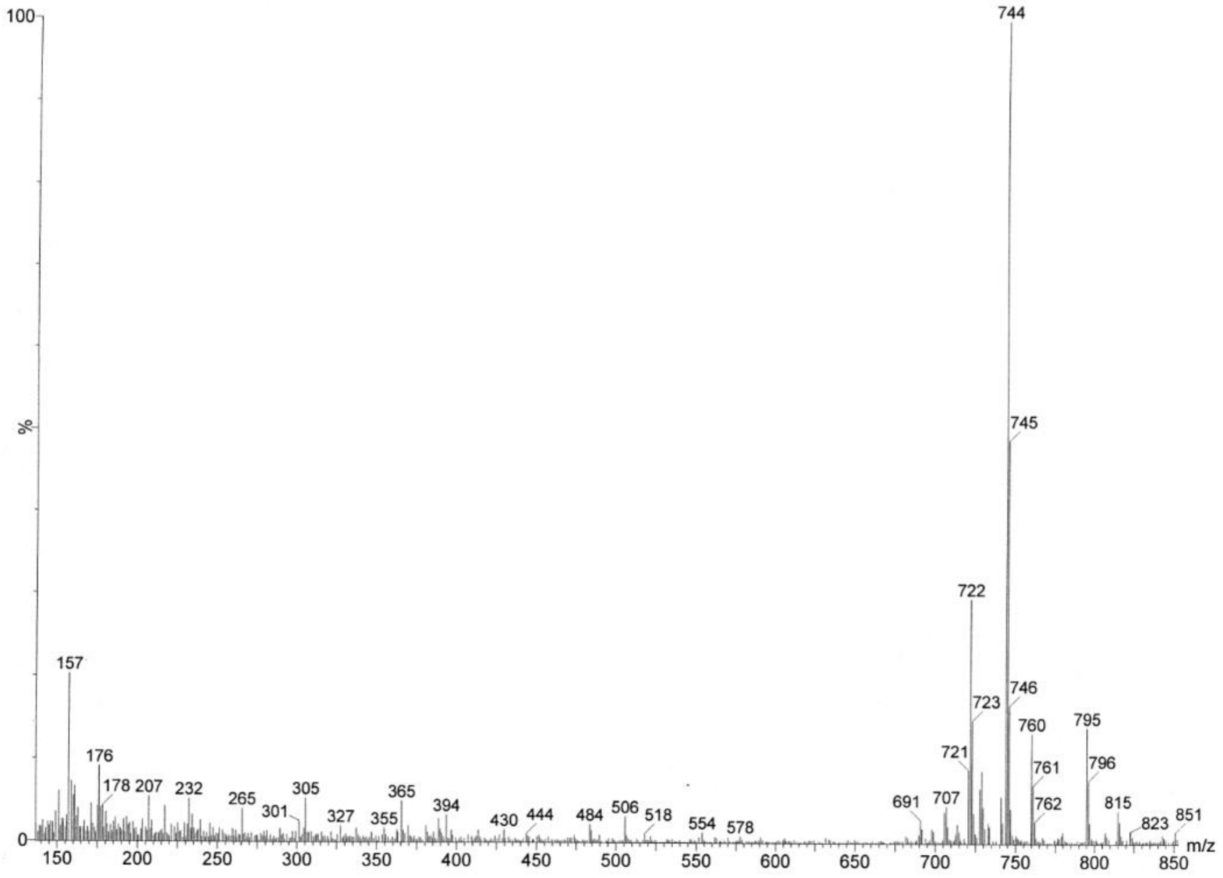

Figure S34. Mass spectrum (ESI +) of **Axle-3** recorded in MeOH.

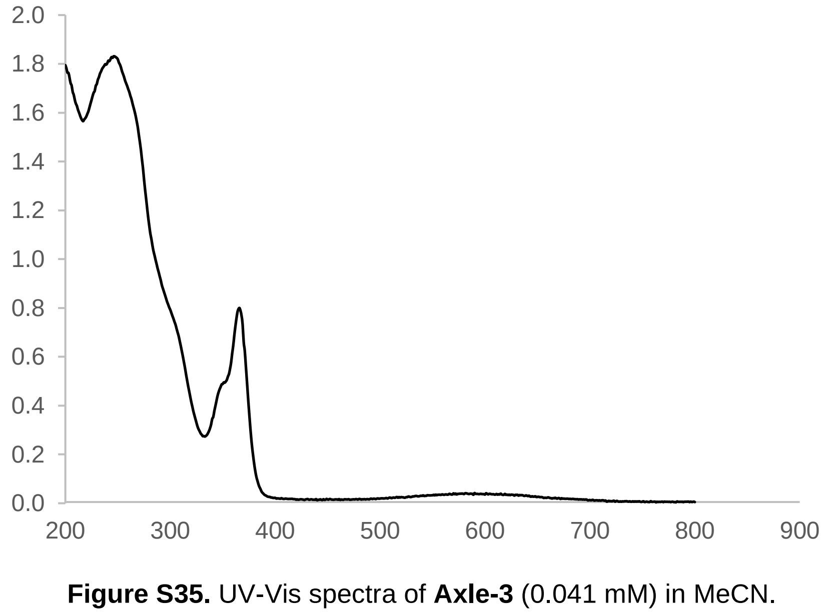

Figure S35. UV-Vis spectra of **Axle-3** (0.041 mM) in MeCN.

EPR spectroscopy.

EPR spectra has been recorded on Bruker-ELEXYS spectrometer by using the following instrument settings: microwave power 0.79 mW, modulation amplitude 0.04 mT, modulation frequency 100 kHz, scan time 180 s, 2K data points. *g*-factors very close to 2.0068 were found in all cases. EPR spectra were transferred to a personal computer for analysis using digital simulations carried out with programs developed in our laboratory and based on a Monte Carlo minimization procedure. [7]

Theory.

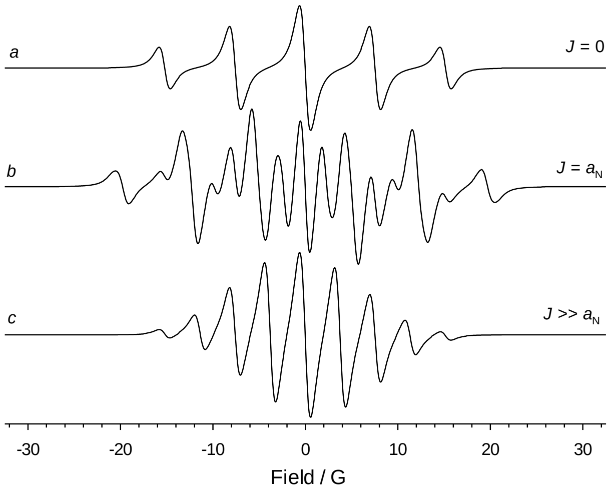

Figure S36. Theoretical spectra for a symmetric bis(nitronyl nitroxide) calculated by using a_N = 7.6 G and (*a*) J = 0; (*b*) $J/g\mu_b = a_N$; (*c*) $J >> a_N$.

J values were determined by simulation of the corresponding experimental spectra by using the following expressions which describe the energy of the four possible transitions for each nuclear spin configuration and their intensities:

Transition	Energy	Intensity
1st	$g_0\mu_0B_0 - \frac{1}{2}J - \frac{1}{2}W + \frac{1}{2}a(M_1 + M_2 + M_3 + M_4)$	$(W - J)/4W$
2nd	$g_0\mu_0B_0 - \frac{1}{2}J + \frac{1}{2}W + \frac{1}{2}a(M_1 + M_2 + M_3 + M_4)$	$(W + J)/4W$
3rd	$g_0\mu_0B_0 + \frac{1}{2}J + \frac{1}{2}W + \frac{1}{2}a(M_1 + M_2 + M_3 + M_4)$	$(W - J)/4W$
4th	$g_0\mu_0B_0 + \frac{1}{2}J - \frac{1}{2}W + \frac{1}{2}a(M_1 + M_2 + M_3 + M_4)$	$(W + J)/4W$

Here $W = \sqrt{\{J^2 + a^2[(M_1 + M_2) - (M_3 + M_4)]^2\}}$ and M_i are nuclear spin configurations of the four interacting nitrogen atoms.

Determinations of jumping rates were obtained by simulation of linewidth variations as function of temperature by using density matrix theory.[8]

.

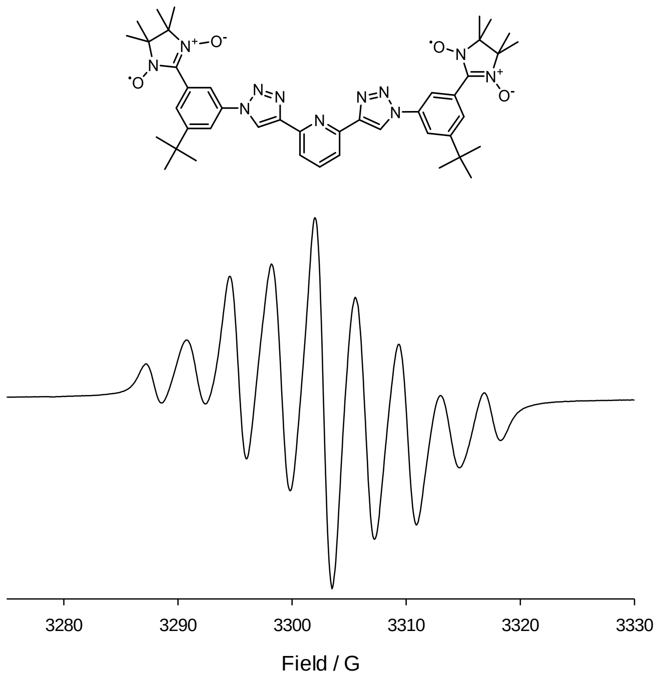

Figure S37. EPR spectrum of **Axle-1** in toluene at room temperature.

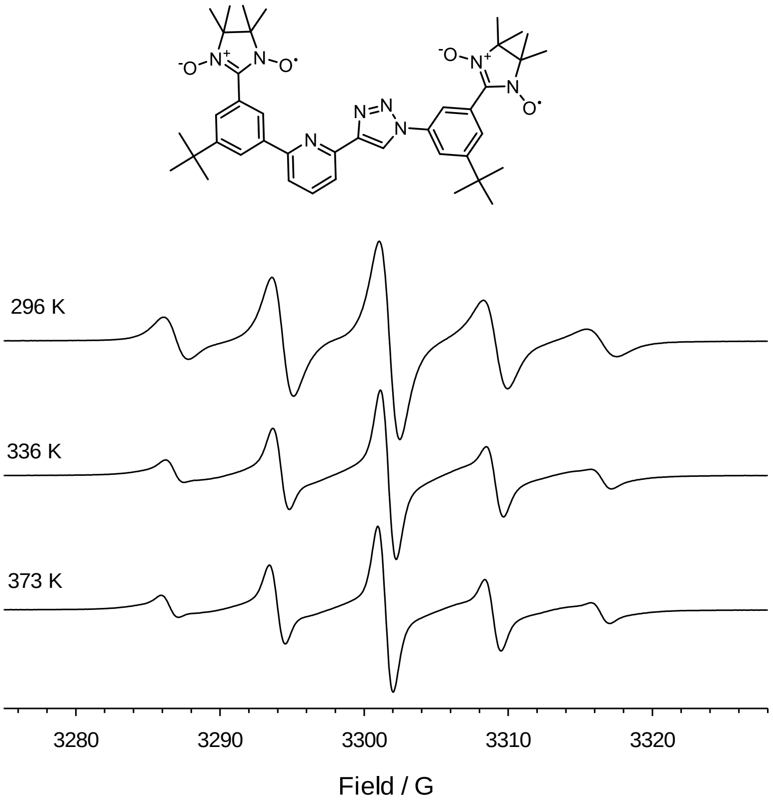

Figure S38. EPR spectra of **Axle-3** in *tert*-butylbenzene at different temperatures.

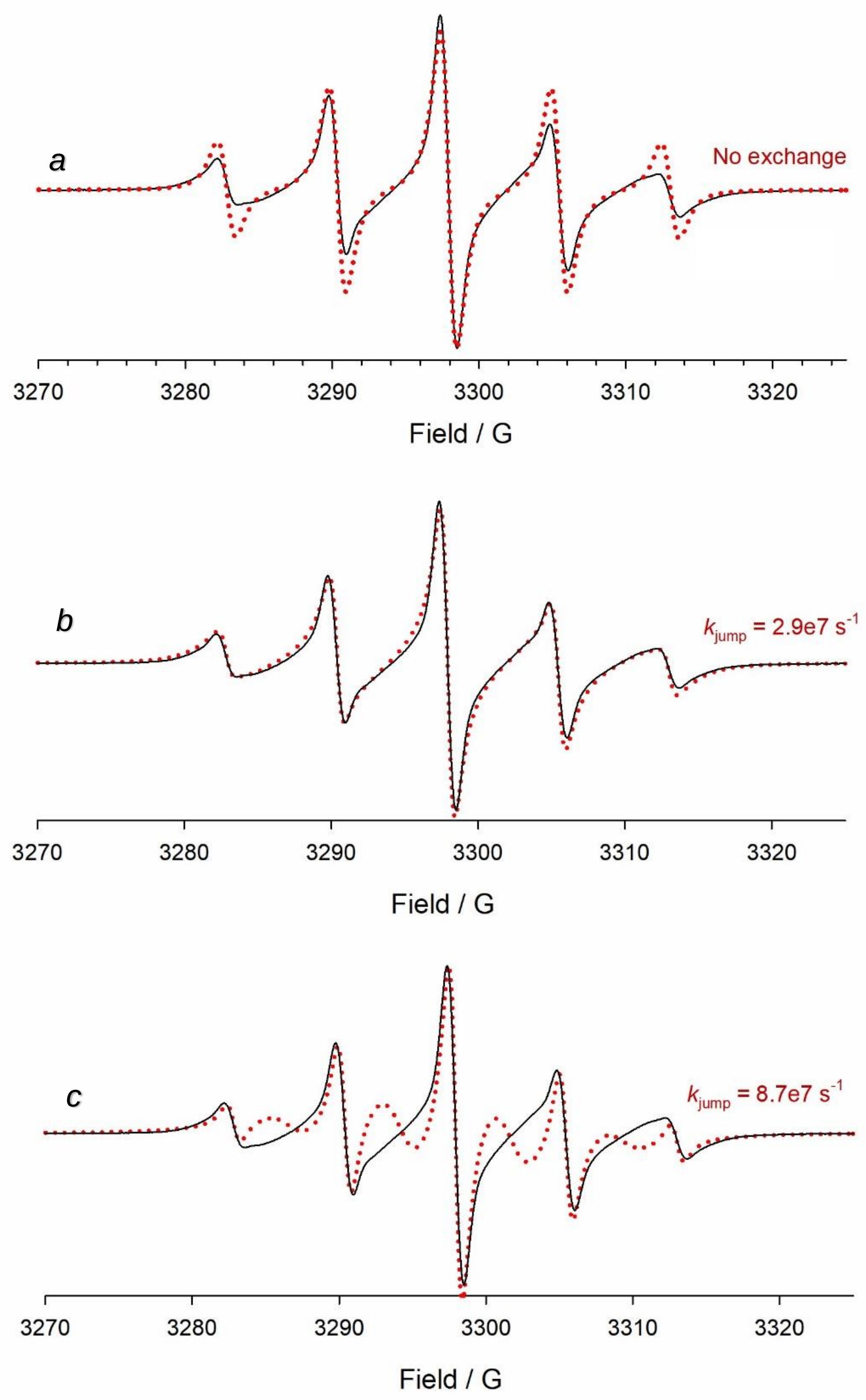

Figure S39. EPR spectrum of **Rotax-3** in MeCN at 298K (black line) and theoretical simulations (red dotted lines) obtained in the absence of exchange (spectrum a), with a rate of exchange equal to 2.9x10^7 s^{-1} (spectrum b) and 8.7x10^7 s^{-1} (spectrum c).

Dynamic simulations. In order to estimate radical-radical distances, Stochastic Dynamic (SD) simulations were carried out with the Macro-Model 7.0 program. In order to use reliable parameters the nitronyl nitroxide moiety bond was modelled as indicated in Figure S58. Extended non-bonded cutoff distances were set to 7 and 12 Å for the van der Waals and electrostatic interactions, respectively. Calculations were performed in *vacuo*. All C-H lengths were held fixed by means of the SHAKE algorithm. The simulations were run at 298 K with time steps of 1.5 fs and an equilibrium time of 3000 ps before each dynamic run. The total simulation time was set to 30000 ps.

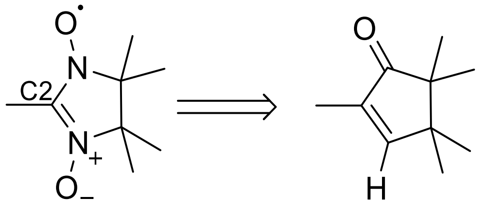

Figure S40. Simplified model employed for dynamic simulation of nitronyl nitroxides.

Table S1. Estimated average distance (Å) between C2-C2`[a] carbon atoms of the two paramagnetic heterocycle units.

Axle	Conformation[b]	Conformation[c]	Distance <*r*>
**1**	*a-a*	*c-c*	7.1
**1**	*a-a*	*c-f* or *f-c*	11.5
**1**	*a-a*	*f-f*	16.0
**2**	*a-a*	*c-c*	7.6
**2**	*a-a*	*c-f*	12.1
**2**	*a-a*	*f-f*	16.5
**2**	*a-s*	*c-c*	15.3
**2**	*a-s*	*c-f*	13.7
**2**	*a-s*	*f-f*	17.5
**2**	*s-s*	*c-c*	17.7
**2**	*s-s*	*c-f*	18.2
**2**	*s-s*	*f-f*	17.2
**3**	-	*c-c*	7.8
**3**	-	*c-f*	11
**3**	-	*f-c*	12
**3**	-	*f-f*	15

[a] See Scheme 1 in the main text for the definition of C2 and C2`. [b] Conformation around the bonds connecting the innermost aromatic ring and the two triazole rings (*a*: *anti*, *s*: *syn*). [c] Conformation around the bonds connecting the triazoles to the aromatic ring bound to the paramagnetic unit (*c*: *close*; *f*: *far*).

1 Gege, C.; Steeneck, C.; Kinzel, O.; Kleymann, G.; Hoffmann, T. Patent: WO 2013/178362 A1, Dec 5, 2013.

2 Li, Q.; Huang, F.; Fan, Y.; Wang, Y.; Li,J.; He, Y.; Jiang, H. *Eur. J. Inorg. Chem*., **2014**, *20*, 3235–3244.

3 De Simone, F.; Saget, T.; Benfatti, F.; Almeida, S.; Waser, J. *Chem. Eur. J.*, **2011**, *17*, 14527-14538.

4 Hirel, C.; Vostrikova, K. E.; Pécaut, J.; Ovcharenko, V. I.; Rey, P. *Chem. Eur. J*., **2001**, *7*, 2007-2014.

5 Bouillon, A.; Lancelot, J.-C.; Sopkova de Oliveira Santos, J.; Collot, V.; Bovyb, P. R.; Rault, S. *Tetrahedron* **2003**, *59*, 10043–10049.

6 Neal, E. A.; Goldup, S. M. *Chem. Sci.*, **2015**, *6*, 2398 – 2404.

7 Romano, F.; Manoni, R.; Franchi, P.; Mezzina, E.; Lucarini, M. *Chem. Eur. J.* **2015**, *21*, 2775–2779.

8 Binsch, G. *J. Am. Chem. Soc.* **1969**, *91*, 1304-1309
